# How Merkel cells transduce mechanical stimuli: A biophysical model of Merkel cells

**DOI:** 10.1371/journal.pcbi.1011720

**Published:** 2023-12-20

**Authors:** Fangtao Mao, Wenzhen Yang

**Affiliations:** Research Center for Humanoid Sensing, Intelligent Perception Research Institute of Zhejiang Lab, Hangzhou, Zhejiang, China; Inria, FRANCE

## Abstract

Merkel cells combine with *Aβ* afferents, producing slowly adapting type 1(SA1) responses to mechanical stimuli. However, how Merkel cells transduce mechanical stimuli into neural signals to *Aβ* afferents is still unclear. Here we develop a biophysical model of Merkel cells for mechanical transduction by incorporating main ingredients such as *Ca*^2+^ and *K*^+^ voltage-gated channels, *Piezo*2 channels, internal *Ca*^2+^ stores, neurotransmitters release, and cell deformation. We first validate our model with several experiments. Then we reveal that *Ca*^2+^ and *K*^+^ channels on the plasma membrane shape the depolarization of membrane potentials, further regulating the *Ca*^2+^ transients in the cells. We also show that *Ca*^2+^ channels on the plasma membrane mainly inspire the *Ca*^2+^ transients, while internal *Ca*^2+^ stores mainly maintain the *Ca*^2+^ transients. Moreover, we show that though *Piezo*2 channels are rapidly adapting mechanical-sensitive channels, they are sufficient to inspire sustained *Ca*^2+^ transients in Merkel cells, which further induce the release of neurotransmitters for tens of seconds. Thus our work provides a model that captures the membrane potentials and *Ca*^2+^ transients features of Merkel cells and partly explains how Merkel cells transduce the mechanical stimuli by *Piezo*2 channels.

## Author summary

Touch is an essential way for humans to sense the physical world. It is necessary to figure out how our tactile system works. However, how Merkel cells convey mechanical stimuli into neural signals and deliver them to *Aβ* afferents is still poorly understood. In this work, we develop a biophysical model of Merkel cells for mechanical transduction. We show that *Ca*^2+^ and *K*^+^ channels on the plasma membrane control the membrane potentials of Merkel cells and further regulate the *Ca*^2+^ transients in the cells. *Ca*^2+^ channels on ER and MT directly contribute to the *Ca*^2+^ transients. Under indentation, the influx of *Ca*^2+^ through *Piezo*2 channels is sufficient to trigger the *Ca*^2+^ transients in Merkel cells and internal *Ca*^2+^ stores inherit to maintain the *Ca*^2+^ transients, which further result in a sustained release of neurotransmitters that activates the *Aβ* afferents. Thus our findings partly explain how Merkel cells combine with *Aβ* afferents to generate an SA1 response to sustained indentation by *Piezo*2 channels.

## Introduction

Tactile end organs transduce various mechanical stimuli into action potential sequences. Among them, Merkel cell-neurite complexes, which mainly produce slowly adapting type 1(SA1) responses [1–3], play an important role in the reception of corners, edges, curvatures of objects, and gentle touch [4, 5].

Merkel cell-neurite complexes generate sustained action potentials with irregular intervals when a sustained mechanical stimulus is applied to the skin [3, 6, 7]. Recent studies found that *Piezo*2 channels, which are expressed in Merkel cells and *Aβ* afferents and only generate a transient current, are necessary for the sustained firing of *Aβ* afferents [7–9]. However, continuous current injections are required for *Aβ* afferents to generate sustained action potentials [3, 10]. Recent theoretical research also thought that rapidly adapting(RA) mechanical-sensitive(MS) channels like *Piezo*2 cannot account for the sustained responses of Merkel cell-neurite complexes [11]. Therefore, how Merkel cells transduce the mechanical stimuli with *Piezo*2 channels has not been well revealed.

Recent studies found that Merkel cells express many molecules that mediate synaptic vesicle release [12–14]. Some studies further checked that Merkel cells release neurotransmitter analogs to *Aβ* afferents [15, 16]. Merkel cells also generate unique *Ca*^2+^ transients under mechanical stimuli [6, 17]. *Ca*^2+^ is an important signal that regulates the release of neurotransmitters [18–20]. Besides, different from other neural cells, Merkel cells do not generate the *Na*^+^-related action potentials but have the *Ca*^2+^ and *K*^+^ dominant membrane potential behaviors [17, 21–23]. Finally, *Piezo*2 channels transport ions into the cell under mechanical stimulus, with a preferential transport of *Ca*^2+^. Therefore, Piezo2 channels, membrane potentials, *Ca*^2+^ transients, and vesicles release together to form a line in Merkel cells to translate the mechanical stimulus. However, a detailed biophysical model of Merkel cells is still lacking. It is still unclear how these parameters participate in the mechanical transduction of Merkel cells.

To address these questions, we establish a biophysical electrophysiological model of Merkel cells. First, we validate our model with experiments of [21–24]. The membrane potentials of Merkel cells under current stimuli vary with the conductances of plasma membrane *Ca*^2+^ channels. The *Ca*^2+^ transients in Merkel cells under high *K*^+^ solutions and hypotonic shock last for tens of seconds. Then, we explore the roles of ion channels on the plasma membrane, endoplasmic reticulum(ER), and mitochondria(MT) in membrane potentials and *Ca*^2+^ transients regulations of Merkel cells. The results show that *Ca*_*v*_1.2 channels mainly contribute to the form of the peak of membrane potentials, while *K*_*v*_1.4 channels inhibit this peak. *BKCa*, *KDR*, and *Ca*_*v*_2.1 channels mainly regulate the steady membrane potentials. *Ca*_*v*_1.2 channels cause the increase of *Ca*^2+^ concentration at the initial time, while *Ca*_*v*_2.1 channels, Ryanodine and *IP*_3_ receptors on the ER increase the duration of *Ca*^2+^ transients. Interestingly, the *Ca*^2+^ channels on MT also lengthen the *Ca*^2+^ transients by satisfying the peak of *Ca*^2+^ transients.

Based on the above results, we further study the mechanical transduction of Merkel cells with *Piezo*2 channels. The results show that Merkel cells generate a continuous neurotransmitter release under a sustained indentation, in which the duration of neurotransmitter release is positively related to indentation depth. This duration lasts for tens of seconds, corresponding to the firing time of Merkel discs under indentation [6, 7, 24]. These results can partly explain how *Piezo*2 channels(RA MS channels) inspire Merkel cells to have the sustained neurotransmitter excitation on *Aβ* afferents for their continuous firing.

## Materials and methods

### Kinetics of ions

Most Merkel cells are globular in shape [25], and some Merkel cells in hair follicles have an irregular shape [24]. To simplify, here we assume the Merkel cell is a sphere with a radius of *r*. Merkel cells contain *Na*^+^, *K*^+^, *Cl*^−^, *Ca*^2+^, and some micro-molecules *A*^−^ with negative charges inside the cell. Generally, cells keep in a near electro-neutral condition [26, 27]. We denote *n*_*Na*_, *n*_*K*_, *n*_*Cl*_, *n*_*Ca*_, and *n*_*A*_ as the mole numbers of *Na*^+^, *K*^+^, *Cl*^−^, *Ca*^2+^, and *A*^−^ inside the cell, respectively. Then their concentrations are *C*_*Na*_ = *n*_*Na*_/(*V* − *V*_*ER*_ − *V*_*MT*_), *C*_*K*_ = *n*_*K*_/(*V* − *V*_*ER*_ − *V*_*MT*_), *C*_*Cl*_ = *n*_*Cl*_/(*V* − *V*_*ER*_ − *V*_*MT*_), *C*_*Ca*_ = *n*_*Ca*_/(*V* − *V*_*ER*_ − *V*_*MT*_), *C*_*A*_ = *n*_*A*_/(*V* − *V*_*ER*_ − *V*_*MT*_), where *V* is the volume of Merkel cells, *V* = 4/3*πr*^3^, *V*_*ER*_ and *V*_*MT*_ are Endoplasmic reticulum and Mitochondria volume. We also set *C*_*Na*,*out*_, *C*_*K*,*out*_, *C*_*Cl*,*out*_, and *C*_*Ca*,*out*_ as *Na*^+^, *K*^+^, *Cl*^−^, and *Ca*^2+^ concentrations in environmental solutions, respectively. Ions inside the cell are regulated by ion channels embedded in the cell membrane.

#### K^+^ channels

The molecular profiling of Merkel cells shows that they express three kinds of voltage-gated *K*^+^ channels: *K*_*v*_4.2, *K*_*v*_1.4, and *K*_*v*_8.1 [12]. However, the study of Yamashita shows there are only two kinds of ion currents of *K*^+^ in Merkel cells, which are similar to currents of *K*_*v*_4.2 and *K*_*v*_1.4 [21]. Further studies elucidated that *K*_*v*_8.1 channels do not generate ion currents directly but regulate other *K*^+^ channels’ activities indirectly [28]. Therefore, here we only consider *K*_*v*_4.2 and *K*_*v*_1.4 channels in Merkel cells.

*K*_*v*_1.4 channels. *K*_*v*_1.4 channels are activated rapidly with the increase of cell membrane potential. A part of the currents inactivates rapidly, while the remaining currents have a slower inactivation [21, 29]. The data for modeling *K*_*v*_1.4 channels was obtained by fitting the data from [29] (Fig B in S1 Appendix),

$$\begin{array}{c} J_{K_{v}1.4}=-g_{K_{v}1.4}m^{4}\left(0.7h_{fast}+0.3h_{slow}\right)\left(V_{m}-E_{K}\right)/F, \end{array}$$ (1)

where $g_{K_{v}1.4}$ is the specific membrane conductance of *K*_*v*_1.4, *V*_*m*_ is the membrane potential, *E*_*K*_ is the equilibrium (or Nernst) potential of *K*^+^, *F* is the Faraday constant. For *m*, *h*_*fast*_, and *h*_*slow*_ [29],

$$\begin{array}{c} \frac{dm}{dt}=\frac{m_{\infty }-m}{\tau_{m}}, \end{array}$$ (2)

$$\begin{array}{c} m_{\infty }=\frac{1}{1+\text{exp}(-\frac{V_{m}+23.12}{11.46})}, \end{array}$$ (3)

$$\begin{array}{c} \tau_{m}=0.6+\text{exp}\left(-\frac{V_{m}+12.02}{25.87}\right), \end{array}$$ (4)

$$\begin{array}{c} \frac{dh_{fast}}{dt}=\frac{h_{\infty }-h_{fast}}{\tau_{h,fast}}, \end{array}$$ (5)

$$\begin{array}{c} h_{\infty }=\frac{1}{1+\text{exp}(\frac{V_{m}+44.36}{2.73})}, \end{array}$$ (6)

$$\begin{array}{c} \tau_{h,fast}=-0.1086\cdot V_{m}+48.67, \end{array}$$ (7)

$$\begin{array}{c} \frac{dh_{slow}}{dt}=\frac{h_{\infty }-h_{slow}}{\tau_{h,slow}}, \end{array}$$ (8)

$$\begin{array}{c} \tau_{h,slow}=17.28. \end{array}$$ (9)

*K*_*v*_4.2 channels. *K*_*v*_4.2 channels are both activated and inactivated rapidly [21, 30]. The model data was obtained from [21] (Fig C in S1 Appendix),

$$\begin{array}{c} J_{K_{v}4.2}=-g_{K_{v}4.2}mh\left(V_{m}-E_{K}\right)/F, \end{array}$$ (10)

where $g_{K_{v}4.2}$ is the specific membrane conductance of *K*_*v*_4.2. For *m*, *h*,

$$\begin{array}{c} \frac{dm}{dt}=\frac{m_{\infty }-m}{\tau_{m}}, \end{array}$$ (11)

$$\begin{array}{c} m_{\infty }=\frac{1}{1+\text{exp}(-\frac{V_{m}-17.66}{22.75})}, \end{array}$$ (12)

$$\begin{array}{c} \tau_{m}=3+2\;\text{exp}\left(-\frac{V_{m}-19.68}{41.23}\right), \end{array}$$ (13)

$$\begin{array}{c} \frac{dh}{dt}=\frac{h_{\infty }-h}{\tau_{h}}, \end{array}$$ (14)

$$\begin{array}{c} h_{\infty }=\frac{1}{1+\text{exp}(\frac{V_{m}+44.36}{2.73})}, \end{array}$$ (15)

$$\begin{array}{c} \tau_{h}=10+\text{exp}\left(-\frac{V_{m}-488}{165.84}\right). \end{array}$$ (16)

In addition to the above two *K*^+^ channels, there are also *Ca*^2+^-activated *K*^+^ channels(*BK*_*Ca*_ channels) and Delayed-rectifier *K* channels(*KDR* channels) in Merkel cells [12, 21, 23]. These two kinds of channels carry the majority of *K*^+^ currents in Merkel cells [21, 23].

*BK*_*Ca*_ channels. *BK*_*Ca*_ channels are activated by membrane potential and have no obvious inactivation behavior. As the intracellular *Ca*^2+^ concentration increases, the curve of the channels’ open probability versus membrane potential has a right shift [23, 31]. Here we take a similar channel model from [32],

$$\begin{array}{c} J_{BK_{Ca}}=-g_{BK_{Ca}}n\left(V_{m}-E_{BK_{Ca}}\right)/F, \end{array}$$ (17)

where $g_{BK_{Ca}}$ is the specific membrane conductance of *BK*_*Ca*_, $E_{BK_{Ca}}$ is the Nernst potential, For *n*,

$$\begin{array}{c} \frac{dn}{dt}=\frac{n_{\infty }-n}{\tau_{n}}, \end{array}$$ (18)

$$\begin{array}{c} pCa=\text{log}\frac{C_{Ca}}{K_{BK_{Ca}}}, \end{array}$$ (19)

$$\begin{array}{c} V_{half}=-43.3pCa-110, \end{array}$$ (20)

$$\begin{array}{c} sf=33.88\;\text{exp}(-{\left(\frac{pCa+5.42}{2.2}\right)}^{2}), \end{array}$$ (21)

$$\begin{array}{c} n_{\infty }=\frac{1}{1+\text{exp}(-\frac{V_{m}-V_{half}}{sf})}, \end{array}$$ (22)

$$\begin{array}{c} \tau_{n}=0.75+5.55\;\text{exp}\left(\frac{V_{m}}{42.91}\right)-0.12V_{m}, \end{array}$$ (23)

where $K_{BK_{Ca}}=1mM$.

*KDR* channels present a slow activation behavior, the parameters are taken from [21] (Fig D in S1 Appendix),

$$\begin{array}{c} J_{KDR}=-g_{KDR}n\left(V_{m}-E_{KDR}\right)/F, \end{array}$$ (24)

where *g*_*KDR*_ is the specific membrane conductance of *KDR*, *E*_*KDR*_ is the Nernst potential, For *n*,

$$\begin{array}{c} \frac{dn}{dt}=\frac{n_{\infty }-n}{\tau_{n}}, \end{array}$$ (25)

$$\begin{array}{c} n_{\infty }=\frac{1}{1+\text{exp}(-\frac{V_{m}+33.3}{8.7})}, \end{array}$$ (26)

$$\begin{array}{c} \tau_{n}=2.2+20\;\text{exp}\left(-{\left(\frac{V_{m}+13.03}{29.55}\right)}^{2}\right). \end{array}$$ (27)

#### Ca^2+^ channels

*Ca*^2+^ plays an important role in Merkel cells’ responses under different stimuli like mechanical stimulation, hypotonic shock, etc [12, 22, 23]. Merkel cells mainly express two kinds of voltage-gated *Ca*^2+^ channels: *Ca*_*v*_1.2 and *Ca*_*v*_2.1 [12].

*Ca*_*v*_1.2 channels. *Ca*_*v*_1.2 channels are L-type(long-lasting) channels, which exhibit a *Ca*^2+^-dependent inactivation [33, 34]. The equations were taken from [32],

$$\begin{array}{c} J_{Ca_{v}1.2}=-g_{Ca_{v}1.2}mh\cdot hCa\left(V_{m}-E_{Ca}\right)/F, \end{array}$$ (28)

where $g_{Ca_{v}1.2}$ is the specific membrane conductance of *Ca*_*v*_1.2, *E*_*Ca*_ is the Nernst potential, for *m*, *h*, and *hCa*,

$$\begin{array}{c} \frac{dm}{dt}=\frac{m_{\infty }-m}{\tau_{m}}, \end{array}$$ (29)

$$\begin{array}{c} m_{\infty }=\frac{1}{1+\text{exp}(-\frac{V_{m}-8.46}{4.26})}, \end{array}$$ (30)

$$\begin{array}{c} \tau_{m}=2.11+3.86\;\text{exp}\left(-2{\left(\frac{V_{m}+10}{16.02}\right)}^{2}\right), \end{array}$$ (31)

$$\begin{array}{c} \frac{dh}{dt}=\frac{h_{\infty }-h}{\tau_{h}}, \end{array}$$ (32)

$$\begin{array}{c} h_{\infty }=\frac{1}{1+\text{exp}(\frac{V_{m}+42.52}{7.48})}, \end{array}$$ (33)

$$\begin{array}{c} \tau_{h}=825.80+637.91\;\text{exp}\left(-2{\left(\frac{V_{m}}{39.75}\right)}^{2}\right), \end{array}$$ (34)

$$\begin{array}{c} hCa=\frac{1}{1+{\left(\frac{C_{Ca}}{K_{hCa}}\right)}^{4}}, \end{array}$$ (35)

where *K*_*hCa*_ = 1*uM*.

*Ca*_*v*_2.1 channels. *Ca*_*v*_2.1 channels are P/Q-type channels [33, 35], which have no obvious inactivation behavior. The equations were taken from [32],

$$\begin{array}{c} J_{Ca_{v}2.1}=-g_{Ca_{v}2.1}n\left(V_{m}-E_{Ca}\right)/F, \end{array}$$ (36)

where $g_{Ca_{v}2.1}$ is the specific membrane conductance of *Ca*_*v*_2.1, *E*_*Ca*_ is the Nernst potential, for *n*,

$$\begin{array}{c} \frac{dn}{dt}=\frac{n_{\infty }-n}{\tau_{n}}, \end{array}$$ (37)

$$\begin{array}{c} n_{\infty }=\frac{1}{1+\text{exp}(-\frac{V_{m}+5.1}{3.1})}, \end{array}$$ (38)

$$\begin{array}{c} \tau_{n}=0.35+5.51\;\text{exp}\left(-2{\left(\frac{V_{m}+9.73}{18.14}\right)}^{2}\right). \end{array}$$ (39)

#### Piezo2 channels

Merkel cells cannot translate mechanical stimuli to neural signals without *Piezo*2 channels, which carry inward currents under the indentation [6, 7]. *Piezo*2 channels are mechanical-sensitive(MS) channels, which can also be opened by the microtubule suction of the membrane [36–38]. Therefore we assume that *Piezo*2 channels’ open probability is regulated by membrane tension. Given that cells have a cortex which is comprised of the cell membrane and a thin, cross-linked actin network lying beneath the membrane, we assume that *Piezo*2 channels open probability is regulated by the cortex stress *σ* [39]. *Piezo*2 channels exhibit a fast activation and a fast inactivation [36, 40]. Besides, *Piezo*2 channels have another inactivation with a much longer time scale [38]. Here we introduce *C*, *O*, *In*, and *h*_*slow*_ to represent the channel closed state, open state, short-time inactivation state, and the state beyond long-time inactivation, respectively. Only channels beyond the long-time inactivation state and open state can transport ions. The data for modeling Piezo2 channels was obtained by fitting the data from [36],

$$\begin{array}{c} J_{Piezo2}=-g_{Piezo2}Oh_{slow}\left(V_{m}-E_{Piezo2}\right)/F, \end{array}$$ (40)

where *J*_*Piezo*2_ is specific membrane conductance of Piezo2 channels, *E*_*Piezo*2_ is the Nernst potential. Under loading,

$$\begin{array}{c} \frac{dC}{dt}=\frac{C_{\infty }-C}{\tau_{C}}, \end{array}$$ (41)

$$\begin{array}{c} \frac{dO}{dt}=-\frac{O}{\tau_{O}}-\frac{C_{\infty }-C}{\tau_{C}}, \end{array}$$ (42)

$$\begin{array}{c} \frac{dIn}{dt}=\frac{O}{\tau_{O}}. \end{array}$$ (43)

Under unloading,

$$\begin{array}{c} \frac{dC}{dt}=-\frac{In_{\infty }-In}{\tau_{In}}, \end{array}$$ (44)

$$\begin{array}{c} \frac{dIn}{dt}=\frac{In_{\infty }-In}{\tau_{In}}. \end{array}$$ (45)

Other parameters are the same all the time.

$$\begin{array}{c} \frac{dh_{slow}}{dt}=\frac{h_{slow,\infty }-h_{slow}}{\tau_{h_{slow}}}, \end{array}$$ (46)

$$\begin{array}{c} C_{\infty }=\frac{1}{1+\text{exp}(\frac{\sigma -\sigma_{s1}}{\sigma_{f1}})}, \end{array}$$ (47)

$$\begin{array}{c} \tau_{C}=0.5+\frac{1.5}{1+\text{exp}(-\frac{\sigma -\sigma_{s2}}{\sigma_{f2}})}, \end{array}$$ (48)

$$\begin{array}{c} \tau_{O}=2.5+\frac{5.5}{1+\text{exp}(-\frac{\sigma -\sigma_{s4}}{\sigma_{f4}})}, \end{array}$$ (49)

$$\begin{array}{c} In_{\infty }=\frac{1}{1+\text{exp}(-\frac{\sigma -\sigma_{s1}}{\sigma_{f1}})}, \end{array}$$ (50)

$$\begin{array}{c} \tau_{In}=0.5+\frac{1.5}{1+\text{exp}(-\frac{\sigma -\sigma_{s2}}{\sigma_{f2}})}, \end{array}$$ (51)

$$\begin{array}{c} h_{slow,\infty }=\frac{1}{1+\text{exp}(-\frac{\sigma -\sigma_{s7}}{\sigma_{f7}})}, \end{array}$$ (52)

$$\begin{array}{c} \tau_{h_{slow}}=\frac{150}{1+\text{exp}(\frac{\sigma -\sigma_{s8}}{\sigma_{f8}})}. \end{array}$$ (53)

**Fig 1:**
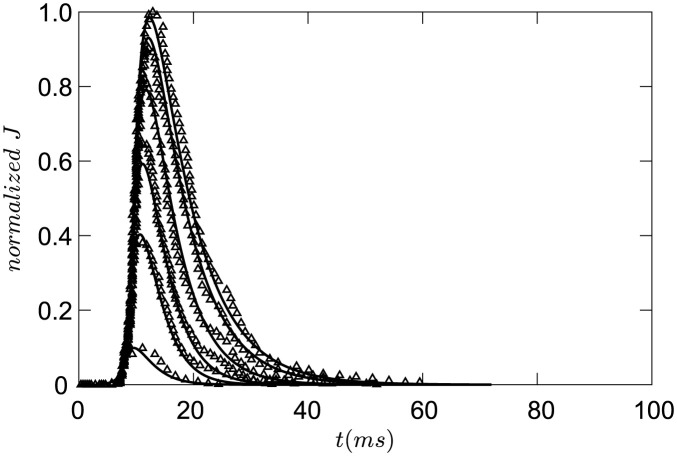
Piezo2 channels. The ions flow generated in *Piezo*2 channels under different indentation depths. triangle line: experimental results from [36], solid line: simulation results(*d*_0_ = 5.8*μm*, *d*_0_ = 6.7*μm*, *d*_0_ = 7.0*μm*, *d*_0_ = 7.3*μm*, *d*_0_ = 7.6*μm*, *d*_0_ = 7.9*μm*).

The parameters of *Piezo*2 channels are listed in Table 1, and the specific descriptions of *Piezo*2 channels are seen in Parameters estimation in S1 Appendix. The currents through *Piezo*2 channels in simulation and experiments are shown in Fig 1.

**Table 1:** Parameters of Piezo2 channels.

Parameter	Description	Value in simulation(ref)
*σ*_*s*1_	Midpoint cortex stress of *C* (*Pa*)	1450 [36]
*σ*_*f*1_	Reference cortex stress of *C* (*Pa*)	120 [36]
*σ*_*s*2_	Midpoint cortex stress of the time constant for *C* (*Pa*)	1450 [36]
*σ*_*f*2_	Reference cortex stress of the time constant for *C* (*Pa*)	40 [36]
*σ*_*s*4_	Midpoint cortex stress of the time constant for *O* (*Pa*)	1450 [36]
*σ*_*f*4_	Reference cortex stress of the time constant for *O* (*Pa*)	100 [36]
*σ*_*s*7_	Midpoint cortex stress of *h*_*slow*_ (*Pa*)	1450 [41]
*σ*_*f*7_	Reference cortex stress of *h*_*slow*_ (*Pa*)	200 [41]
*σ*_*s*8_	Midpoint cortex stress of the time constant for *h*_*slow*_ (*Pa*)	1450 [41]
*σ*_*f*8_	Reference cortex stress of the time constant for *h*_*slow*_ (*Pa*)	2000 [41]
*g*_*Piezo*2_	Specific membrane conductance of Piezo2(*mS*/*cm*^2^)	3 [7]
*E*_*Piezo*2_	Nernst potential (mV)	6 [36, 40]

*Piezo*2 channels are non-selective anion channels [36, 40]. All *Ca*^2+^, *K*^+^, and *Na*^+^ could flow across *Piezo*2 channels. But *Ca*^2+^ has the highest priority [36, 40]. Therefore, we assumed that the currents across Piezo2 channels are *Ca*^2+^ currents.

Besides these channels above, Merkel cells have a passive electrical property. We take *Na*^+^, *K*^+^, *Cl*^−^, and *Ca*^2+^ leak channels into consideration. These leak ions flow can be written as

$$\begin{array}{c} J_{Na,leak}=-\frac{1}{F}g_{Na,leak}\left(V_{m}-E_{Na}\right), \end{array}$$ (54)

$$\begin{array}{c} J_{K,leak}=-\frac{1}{F}g_{K,leak}\left(V_{m}-E_{K}\right), \end{array}$$ (55)

$$\begin{array}{c} J_{Cl,leak}=\frac{1}{F}g_{Cl,leak}\left(V_{m}-E_{Cl}\right), \end{array}$$ (56)

$$\begin{array}{c} J_{Ca,leak}=\frac{1}{F}g_{Ca,leak}\left(V_{m}-E_{Ca}\right), \end{array}$$ (57)

where *g*_*i*,*leak*_ and *E*_*i*_ are the specific membrane conductance and the equilibrium (or Nernst) potential of *i*, $E_{i}=-\frac{RT}{z_{i}F}\;\text{ln}\;\frac{C_{i}}{C_{i,out}}$, *R* is the gas constant, *T* is the thermodynamic temperature, *z*_*i*_ is the valence of ions, *i* = *Na*^+^, *K*^+^, *Cl*^−^, *Ca*^2+^.

#### Pumps, cotransporters, and exchangers

Similar to most cells, Merkel cells also contain ion pumps, cotransporters, and exchangers to keep the ions’ balance inside the cell. Here we take *Na*^+^/*K*^+^ pumps, *Na*^+^/*K*^+^/*Cl*^−^ cotransporters, *K*^+^/*Cl*^−^ cotransporters, *Na*^+^/*Ca*^2+^ exchangers, and *Ca*^2+^ pumps on the plasma membrane into consideration.

*Na*^+^/*K*^+^ pumps, which transport two *K*^+^ into the cell and three *Na*^+^ out of the cell once by consuming energy, keep a high *K*^+^ concentration and a low *Na*^+^ concentration in the cell [42, 43]. The intracellular concentrations of *Na*^+^ and *K*^+^ affect the rate of *Na*^+^/*K*^+^ pumps [44, 45],

$$\begin{array}{c} J_{NaKpump}=P_{NaKpump}\frac{1}{{\left(1+K_{Na,NaK}/C_{Na}\right)}^{3}}\frac{1}{{\left(1+C_{K}/K_{K,NaK}\right)}^{2}}, \end{array}$$ (58)

where *P*_*NaKpump*_ is rate constant. *K*_*Na*,*NaK*_ and *K*_*K*,*NaK*_ are constants, *K*_*Na*,*NaK*_ = 10*mM*, *K*_*K*,*NaK*_ = 140*mM* [46].

*Na*^+^/*K*^+^/*Cl*^−^ cotransporters, which transport one *Na*^+^, one *K*^+^, and two *Cl*^−^ in the same direction once, mainly loading *Cl*^−^ into the cell [47–49]. Their flow is controlled by three ions concentrations [27],

$$\begin{array}{c} J_{NKCC1}=P_{NKCC1}\left(C_{Na,out}C_{K,out}{C_{Cl,out}}^{2}-C_{Na}C_{K}{C_{Cl}}^{2}\right), \end{array}$$ (59)

where *P*_*NKCC*1_ is the rate constant.

*K*^+^/*Cl*^−^ cotransporters, which transport one *K*^+^ and one *Cl*^−^ in the same direction once, mainly extruding *Cl*^−^ from inside the cell [48]. Their flow was regulated by the *Cl*^−^ and *K*^+^ concentrations across the membrane [50–52],

$$\begin{array}{c} J_{KCC2}=P_{KCC2}\frac{C_{Cl}}{C_{Cl,out}}\frac{C_{K}}{C_{K,out}}, \end{array}$$ (60)

where *P*_*KCC*2_ is the rate constant.

Plasma membrane *Ca*^2+^ pumps and *Na*^+^/*Ca*^2+^ exchangers both help to eliminate *Ca*^2+^ from inside the cell [53, 54]. A general model of *Ca*^2+^ pumps was used [55],

$$\begin{array}{c} J_{Capump}=P_{Capump}\frac{C_{Ca}^{2}}{C_{Ca}^{2}+K_{Capump}^{2}}, \end{array}$$ (61)

where *P*_*Capump*_ is the rate constant, *K*_*Capump*_ is constant, *K*_*Capump*_ = 0.3*uM*.

*Na*^+^/*Ca*^2+^ exchangers exhibit complex dynamic behavior. They are motivated by membrane potential, *Ca*^2+^ and *Na*^+^ concentrations. Under steady state, *Na*^+^/*Ca*^2+^ exchangers transport *Ca*^2+^ outside the cell. When the cell is stimulated by currents or membrane voltage, the direction of *Na*^+^/*Ca*^2+^ exchangers flow will be reversed [56, 57]. The dynamic equation of *Na*^+^/*Ca*^2+^ exchangers can be written as [57],

$$\begin{array}{c} \begin{array}{cc} J_{Cana} & =P_{Cana}\frac{1}{K_{mNa}^{3}+C_{Na,out}^{3}}\frac{1}{K_{mCa}+C_{Ca,out}}\frac{1}{1+ksat\cdot exp\left(\eta -1\right)V_{m}F/\left(RT\right))}\\ & \cdot \text{exp}\left(\eta V_{m}\frac{F}{RT}\right)C_{Na}^{3}C_{Ca,out}-\text{exp}\left(\left(\eta -1\right)V_{m}\frac{F}{RT}\right)C_{Na,out}^{3}C_{Ca}, \end{array} \end{array}$$ (62)

where *P*_*Cana*_ is the rate constant, *K*_*mNa*_ and *K*_*mCa*_ are constants, *K*_*mNa*_ = 87.5*mM*, *K*_*mCa*_ = 0.5*uM*, *η* = 0.1, *ksat* = 0.35 [57].

### Internal *Ca*^2+^ dynamics

Intracellular *Ca*^2+^ sources also play an important role in *Ca*^2+^ regulation in Merkel cells. The elimination of internal *Ca*^2+^ store greatly reduces the *Ca*^2+^ transients [22, 23].

Endoplasmic reticulum and Mitochondria are the main internal *Ca*^2+^ stores. There are main three channels on the Endoplasmic reticulum (ER) membrane including *Ca*^2+^ ATPase pump, Inositol 1,4,5-trisphosphate receptor (*IP*_3_ receptor), and Ryanodine receptor. *Ca*^2+^ ATPase pumps consume energy to actively load *Ca*^2+^ into endoplasmic reticulum, and their rate increase with cytoplasmic *Ca*^2+^ concentration [58, 59]

$$\begin{array}{c} J_{pump,ER}=P_{pump,ER}\frac{C_{Ca}^{2}}{C_{Ca}^{2}+K_{ERpump}^{2}}, \end{array}$$ (63)

where *P*_*pump*,*ER*_ is the rate constant, *K*_*ERpump*_ is the dissociation constant, and their values are listed in Table 2.

**Table 2:** Parameters of internal Ca^2+^ channels.

Parameter	Description	Value in simulation(ref)
*K*_*ERpump*_	Dissociation constant (*uM*)	0.1 [59]
*K*_*s*,*RYR*_	Midpoint *Ca*^2+^ concentration for RYR (*uM*)	0.3 (Tuned)
*K*_*f*,*RYR*_	Reference *Ca*^2+^ concentration for RYR (*uM*)	0.04 (Tuned)
$K_{s1,IP_{3}}$	Midpoint *Ca*^2+^ concentration for *IP*_3_ receptors (*uM*)	0.4 [60, 61]
$K_{f1,IP_{3}}$	Reference *Ca*^2+^ concentration for *IP*_3_ receptors (*uM*)	0.04 [60, 61]
$K_{s2,IP_{3}}$	Midpoint *Ca*^2+^ concentration for *IP*_3_ receptors (*uM*)	0.6 [60, 61]
$K_{f2,IP_{3}}$	Reference *Ca*^2+^ concentration for *IP*_3_ receptors (*uM*)	0.04 [60, 61]
$K_{s,preIP_{3}}$	Midpoint *preIP*3 concentration (*uM*)	5 [63]
$K_{f,preIP_{3}}$	Reference *preIP*3 concentration (*uM*)	1 [63]
*τ*_*m*_	time constant (*ms*)	10000 [22, 23]
*τ*_*h*_	time constant (*ms*)	20000 [22, 23]
$K_{IP_{3}}$	Dissociation constant (*uM*)	3 [60]
$K_{IP_{3}Ca}$	Dissociation constant (*uM*)	0.5 [63]
*S*_*ER*_	Surface of ER (*um*^2^)	150 [66]
*V*_*ER*_	Volume of ER (*um*^3^)	100 [66]
$k_{preIP_{3}}$	rate constant (*mol*/(*cm*^3^ ⋅ *ms*))	10^−12^ (Tuned)
$k_{IP_{3}}$	rate constant (1/*ms*)	4 × 10^−5^ [63]
$k_{dIP_{3}}$	rate constant (1/*ms*)	2 × 10^−5^ [63]
*K*_*MCU*_	Dissociation constant (*uM*)	0.6 [67]
*K*_*MNCX*_	Dissociation constant (*uM*)	1 (Tuned)
*S*_*MT*_	Surface of mitochondria (*um*^2^)	150 [66]
*V*_*MT*_	Volume of mitochondria (*um*^3^)	10 [66]

Ryanodine receptors and *IP*_3_ receptors involve in *Ca*^2+^-induced *Ca*^2+^ release in Merkel cells [22, 23]. The *Ca*^2+^ flux by Ryanodine receptor is defined by the equation,

$$\begin{array}{c} J_{RYR}=\{\begin{array}{cc} P_{RYR}\frac{1}{1+\text{exp}(-\frac{C_{Ca}-K_{s,RYR}}{K_{f,RYR}})}\left(C_{Ca,ER}-C_{Ca}\right), & C_{Ca}>K_{s,RYR}\\ 0, & C_{Ca}\leq K_{s,RYR} \end{array} \end{array}$$ (64)

where *P*_*RYR*_ is the rate constant, *K*_*s*,*RYR*_ and *K*_*f*,*RYR*_ are constant.

*IP*_3_ receptors have three sites for the combination of *Ca*^2+^ and *IP*_3_. One site is for activated *Ca*^2+^, and one site is for inhibitory *Ca*^2+^ [60]. It means that the increase of *Ca*^2+^ concentration from a low level, the combination of *Ca*^2+^ to *IP*_3_ receptors activates the receptor, as the *Ca*^2+^ concentration increase to a very high level, the receptor will be inhibited [61]. We introduce *m* and *h* to represent the activation and inhibitory role of *Ca*^2+^ on *IP*_3_ receptor,

$$\begin{array}{c} \frac{dm}{dt}=\frac{m_{\infty }-m}{\tau_{m}}, \end{array}$$ (65)

$$\begin{array}{c} \frac{dh}{dt}=\frac{h_{\infty }-m}{\tau_{h}}, \end{array}$$ (66)

where *τ*_*m*_ and *τ*_*h*_ are time constants.

$$\begin{array}{c} m_{\infty }=\frac{1}{1+\text{exp}(-\frac{C_{Ca}-K_{s1,IP_{3}}}{K_{f1,IP_{3}}})}, \end{array}$$ (67)

$$\begin{array}{c} h_{\infty }=\frac{1}{1+\text{exp}(\frac{C_{Ca}-K_{s2,IP_{3}}}{K_{f2,IP_{3}}})}. \end{array}$$ (68)

The remaining site is for *IP*_3_, and the increase of *IP*_3_ concentration enhances the currents of *IP*_3_ receptors [61]. Thus we assume that *IP*_3_ receptors’ open probability equals to

$$\begin{array}{c} J_{ER}=\left(P_{leak}+P_{IP_{3}}\frac{C_{IP_{3}}^{3}}{C_{IP_{3}}^{3}+K_{IP_{3}}^{3}}mh\right)\left(C_{Ca,ER}-C_{Ca}\right), \end{array}$$ (69)

where *P*_*leak*_ and $P_{IP_{3}}$ are rate constants, *P*_*leak*_ represents the leak flow of *Ca*^2+^ on ER. $K_{IP_{3}}$ is the dissociation constant, *C*_*Ca*,*ER*_ is the concentration of *Ca*^2+^ in ER.

*IP*_3_ mobilizes *Ca*^2+^ from intracellular stores through the *IP*_3_ receptors [62], and itself also takes a dynamic regulation in a single cell [63]. Generally, the entry of *Ca*^2+^ through *Ca*^2+^ channels on the membrane cause the hydrolyzation of phosphatidylinositol 4,5-bisphosphate (*PIP*_2_), which produces *IP*_3_ [64]. Then *IP*_3_ will convert to other matters even though the *Ca*^2+^ concentration is still high [63]. Therefore, we assume the production rate of *IP*_3_ increases with the *Ca*^2+^ concentration and the conversion rate of *IP*_3_ depends on its concentration,

$$\begin{array}{c} \frac{dC_{IP_{3}}}{dt}=k_{IP_{3}}\frac{C_{Ca}^{2}}{C_{Ca}^{2}+K_{IP_{3}Ca}}C_{preIP_{3}}-k_{dIP_{3}}C_{IP_{3}}, \end{array}$$ (70)

where $k_{IP_{3}}$ and $k_{dIP_{3}}$ are the rate constants, $K_{IP_{3}Ca}$ is the dissociation constant, $c_{preIP_{3}}$ is the concentration of *IP*_3_ precursors like *IP*_2_ [64].

*preIP*_3_ will be also replenished after consumption, and keep a rather stable state [65]. So we assume that *preIP*_3_ has a production rate related to its concentration. Then the change of *preIP*_3_ will be

$$\begin{array}{c} \frac{dC_{preIP_{3}}}{dt}=\frac{k_{preIP_{3}}}{1+\text{exp}(\frac{C_{preIP_{3}}-K_{s,preIP_{3}}}{K_{f,preIP_{3}}})}-k_{IP3}\frac{C_{Ca}^{2}}{C_{Ca}^{2}+K_{IP_{3}Ca}^{2}}C_{preIP_{3}}. \end{array}$$ (71)

According to the above three *Ca*^2+^ channels, the dynamic equation of *Ca*^2+^ in ER will be

$$\begin{array}{c} \frac{dn_{Ca,ER}}{dt}=S_{ER}\left(-J_{ER}-J_{CICR}+J_{Capump,ER}\right). \end{array}$$ (72)

*C*_*Ca*,*ER*_ = *n*_*Ca*,*ER*_/*V*_*ER*_.

There are Mitochondrial uniporter(MCU) and mitochondrial *Na*^+^/*Ca*^2+^ exchanger (MNCX) on the mitochondrial membrane. MCU takes up *Ca*^2+^ into Mitochondria while MNCX releases *Ca*^2+^ from mitochondria to the cytoplasm. The Mitochondrial uniporter dynamics is controlled by the cytoplasm *Ca*^2+^ concentration [32],

$$\begin{array}{c} J_{MCU}=P_{MCU}\frac{C_{Ca}^{2.3}}{C_{Ca}^{2.3}+K_{MCU}^{2.3}}, \end{array}$$ (73)

where *P*_*MCU*_ is the rate constant, *K*_*MCU*_ is the dissociation constant.

The dynamics of mitochondrial *Na*^+^/*Ca*^2+^ exchanger are controlled by mitochondrial *Ca*^2+^ concentration [32],

$$\begin{array}{c} J_{MNCX}=P_{MNCX}\frac{C_{Ca,MT}}{C_{Ca,MT}+K_{MNCX}}, \end{array}$$ (74)

where *P*_*MNCX*_ is the rate constant, *K*_*MNCX*_ is the dissociation constant. *c*_*Ca*,*MT*_ is mitochondrial *Ca*^2+^ concentration, *c*_*Ca*,*MT*_ = *n*_*Ca*,*MT*_/*V*_*MT*_. The dynamic equation of *n*_*Ca*,*MT*_ will be

$$\begin{array}{c} \frac{dn_{Ca,MT}}{dt}=S_{MT}/\left(1+\beta_{MT}\right)\left(-J_{MNCX}+J_{MCU}\right), \end{array}$$ (75)

where *S*_*MT*_ is the mitochondria surface, *β*_*MT*_ = 0.3, which represents the buffer role for *Ca*^2+^ of mitochondria membrane [32].

According to the above ion channels, the ions change inside the cell can be written as

$$\begin{array}{c} \frac{dn_{Na}}{dt}=S_{ref}\left(-3J_{NaKpump}+J_{NKCC1}-3J_{Cana}+J_{Na,leak}\right), \end{array}$$ (76)

$$\begin{array}{c} \frac{dn_{K}}{dt}=S_{ref}\left(J_{K_{v}1.4}+J_{K_{v}4.2}+J_{KDR}+J_{BCa}+2J_{pump}+J_{NKCC1}-J_{KCC2}+J_{K,leak}\right), \end{array}$$ (77)

$$\begin{array}{c} \frac{dn_{Cl}}{dt}=S_{ref}\left(2J_{NKCC1}-J_{KCC2}+J_{Cl,leak}\right), \end{array}$$ (78)

where *S*_*ref*_ is the reference surface area of Merkel cell.

$$\begin{array}{c} \begin{array}{cc} \frac{dn_{Ca}}{dt} & =S_{ref}\left(J_{Ca_{v}1.2}+J_{Ca_{v}2.1}-J_{Capump}+J_{Cana}-J_{piezo2}+J_{Ca,leak}\right)\\ & +S_{ER}\left(J_{ER}+J_{CICR}-J_{Capump,ER}\right)+S_{MT}/\left(1+\beta_{MT}\right)\left(J_{MNCX}-J_{MCU}\right), \end{array} \end{array}$$ (79)

The membrane potentials of Merkel cells are regulated by currents across total ion channels. The *Na*^+^/*K*^+^ pumps transport two *K*^+^ into the cell and three *Na*^+^ out of the cell, so there is one charge out of the cell once. The cotransporters transport a *Na*^+^, a *K*^+^, and two *Cl*^−^ in the same direction once, so the total discharge is zero. The *Na*^+^/*Ca*^2+^ exchangers transduce three *Na*^+^ into the cell and one *Ca*^2+^ out of the cell once, so there is one charge out of the cell once. Then the dynamic equation of *V*_*m*_ is

$$\begin{array}{c} \begin{array}{cc} C_{m}\frac{dV_{m}}{dt} & =(J_{K_{v}1.4}+J_{K_{v}4.2}+J_{KDR}+J_{BCa}+2J_{Ca_{v}1.2}+2J_{Ca_{v}2.1}+2J_{piezo2}\\ & -J_{NaKpump}-J_{Cana}-2J_{Capump}+J_{K,leak}+J_{Na,leak}-J_{Cl,leak}+2J_{Ca,leak})F, \end{array} \end{array}$$ (80)

where *C*_*m*_ is the specific membrane capacitance.

### Volume change

The cell volume *V* change is dependent on the flow of water across the cell membrane [39]

$$\begin{array}{c} \frac{dV}{dt}=S\cdot J_{water}, \end{array}$$ (81)

where *S* is the cell surface area, *S* = 4*πr*^2^, *J*_*water*_ is water flux, it is controlled by the hydrostatic pressure difference and the osmotic pressure difference across the membrane [39]

$$\begin{array}{c} J_{water}=-\alpha \left(\Delta P-\Delta \Pi \right), \end{array}$$ (82)

where *α* is a rate constant *α* = 10^−9^*cm* ⋅ *ms*^−1^ ⋅ *Pa*^−1^, ΔΠ is the osmotic pressure difference across the membrane, which can be described as

$$\begin{array}{c} \Delta \Pi =(C_{Na}+C_{K}+C_{Cl}+C_{Ca}+C_{A}-C_{Na,out}-C_{K,out}-C_{Cl,out}-C_{Ca,out})RT. \end{array}$$ (83)

Cells have a cortex which is comprised of the cell membrane and a thin, cross-linked actin network lying beneath the membrane. We take the cortex as an elastic layer, the cortex stress satisfies [39]

$$\begin{array}{c} \sigma =\frac{K}{2}\left(\frac{S}{S_{ref}}-1\right)-\sigma_{a}, \end{array}$$ (84)

where *K* is the cortex elastic modulus, *K* = 6000*Pa*, *S*_*ref*_ represents the cell surface under no stress, *σ*_*a*_ is the active contraction stress produced by the actin network, *σ*_*a*_ = −100*Pa*. For a spherical cell, the relationship between the cortex stress and hydrostatic pressure satisfies [39]

$$\begin{array}{c} \Delta P=\frac{2h_{c}\sigma }{r}, \end{array}$$ (85)

where *h*_*c*_ is the cortex thickness, *h*_*c*_ = 0.5*μm* [39].

## Results

### Validation of the model

There are three main stimuli to study the properties of Merkel cells, which are current pulses, high *K*^+^ solutions stimuli, and hypotonic shocks [7, 21–23]. We consider these three kinds of situations below.

#### Current pulses

**Fig 2:**
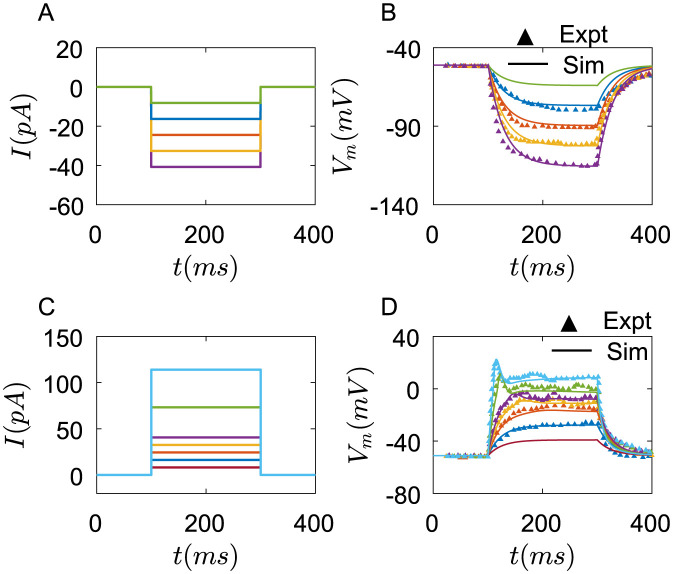
The membrane potential dynamics of one Merkel cell under the rectangular current pulses. (A) Negetive current pulses step in 8.14pA (experimental current step in 10pA). (B) Membrane potentials change under negative current pulses. The solid lines are simulation results, and the triangles are experimental results from [21]. (C) Positive current pulses step in 8.14pA, Green line: 9 × 8.14pA, blue line: 14 × 8.14pA. (D) Membrane potentials change under positive current pulses. The solid lines are the simulation results, and the triangles are experimental results from [21]. The parameters of ion channels are seen in Table 3.

The rectangular negative current pulses induce the membrane potential dynamics of Merkel cells(Fig 2A and 2B), and the parameters of ion channels are seen in Table 3. The solid lines are simulation results and the triangle lines are experimental results from [21]. The results show that Merkel cells have almost passive responses to negative current pulses(Fig 2B).

**Table 3:** Parameters differences of ion channels in Figs 2 and 3.

Parameter	Value in Fig 2	Value in Fig 3
*C*_*m*_(*uF*/*μm*^2^)	4 × 10^−8^ [21]	2 × 10^−8^ [7]
*g*_*K*_*v*_1.4_(*mS*/*cm*^2^)	0.25 [21, 23]	4 [21, 23]
*g*_*K*_*v*_4.2_(*mS*/*cm*^2^)	0.2 [21, 23]	0.2 [21, 23]
$g_{BK_{Ca}}\left(mS/cm^{2}\right)$	0.18 [21, 23]	0.3 [21, 23]
*g*_*KDR*_(*mS*/*cm*^2^)	0.01 [21, 23]	0.01 [21, 23]
$g_{Ca_{v}1.2}(mS/cm^{2})$	2 [21, 23]	0.8 [21, 23]
$g_{Ca_{v}2.1}(mS/cm^{2})$	0.1 × 10^−2^ [21]	0.05 × 10^−2^ [21]
*g*_*Piezo*2_(*mS*/*cm*^2^)	3 [7]	3 [7]
*g*_*Na*,*leak*_(*mS*/*cm*^2^)	0.043(Tuned)	0.045(Tuned)
*g*_*K*,*leak*_(*mS*/*cm*^2^)	0.09(Tuned)	0.09(Tuned)
*g*_*Cl*,*leak*_(*mS*/*cm*^2^)	0.5 × 10^−2^(Tuned)	0.5 × 10^−2^(Tuned)
*g*_*Ca*,*leak*_(*mS*/*cm*^2^)	0.2 × 10^−4^(Tuned)	0.2 × 10^−4^(Tuned)
*P*_*NaKpump*_(*mol*/(*cm*^2^ ⋅ *ms*))	0.37312 × 10^−12^ [68]	0.1696 × 10^−12^ [68]
*P*_*NKCC*1_(*cm*^10^/(*mol*^3^ ⋅ *ms*)	0.24 × 10^2^(Tuned)	0.24 × 10^2^(Tuned)
*P*_*KCC*2_(*mol*/(*cm*^2^ ⋅ *ms*))	0.25 × 10^−14^ [48, 69]	0.25 × 10^−14^ [48, 69]
*P*_*Capump*_(*mol*/(*cm*^2^ ⋅ *ms*))	0.3 × 10^−15^ [22]	150 * 0.3 × 10^−15^ [24]
*P*_*Cana*_(*mol*/(*cm*^2^ ⋅ *ms*))	0.1 × 10^−12^ [22, 23]	0.1 × 10^−12^ [22, 23]
*P*_*pump*,*ER*_(*mol*/(*cm*^2^ ⋅ *ms*))	0.7 × 10^−17^ [22, 23]	0.7 × 10^−17^ [22, 23]
*P*_*RYR*_(*cm*/*ms*)	8.5 [22, 23]	8.5 [22, 23]
*P*_*leak*_(*cm*/*ms*)	0.7 × 10^−3^(Tuned)	0.7 × 10^−3^(Tuned)
$P_{IP_{3}}\left(cm/ms\right)$	1.75 [22, 23]	1.75 [22, 23]
*P*_*MCU*_(*mol*/(*cm*^2^ ⋅ *ms*))	0.5 × 10^−15^(Tuned)	0.5 × 10^−15^(Tuned)
*P*_*MNCX*_(*mol*/(*cm*^2^ ⋅ *ms*))	0.1 × 10^−15^(Tuned)	0.1 × 10^−15^(Tuned)

However, the membrane properties of Merkel cells under positive current pulses are rather different. As shown in Fig 2C and 2D, when the amplitude of the current pulses is small, the membrane potential dynamics of Merkel cells are still like a passive response. As the currents increase(green and wathet blue line), the membrane potentials rise rapidly to a peak, then decrease and gradually stabilize. Unlike most other sensory cells or afferents, Merkel cells do not generate action potentials [7, 21].

**Fig 3:**
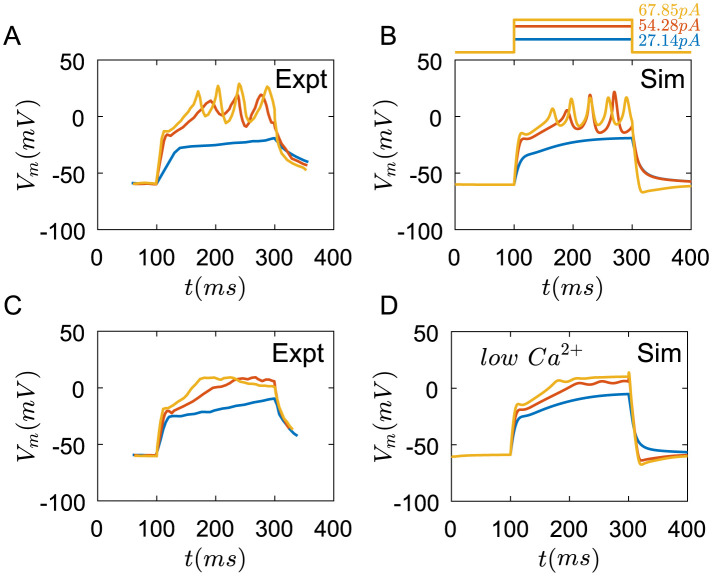
The membrane potential dynamics of one Merkel cell with different properties under the rectangular current pulses. (A) The changes of membrane potential of experimental results from [24]. (B) Membrane potentials change under current pulses in simulation, (C) The changes of membrane potential under the condition of reduced *Ca*^2+^ concentration in external solutions from [24]. (D) Membrane potentials change under current pulses in simulation at *C*_*Ca*,*out*_ = 5*uM*. blue line: 27.14pA, red line: 54.28pA, yellow line: 67.85pA. The parameters of ion channels are seen in Table 3.

Some Merkel cells exhibit more complex membrane potential dynamics [24] (Fig 3A). By changing the specific membrane conductance of *Ca*^2+^ and *K*^+^ channels and rate constants of some pumps on the membrane, we can get similar results. As shown in Fig 3B, the membrane potentials of Merkel cells rapidly reach a step, then fire. *Ca*^2+^ plays an important role in this membrane potential dynamics. If the *Ca*^2+^ concentration is reduced in the external solutions, the fire of membrane potentials disappears(Fig 3C and 3D).

Merkel cells not only have membrane potential regulation but also show *Ca*^2+^ dynamic behaviors. The common stimuli which elicit *Ca*^2+^ in experiments are high *K*^+^ solutions and hypotonic shock.

#### High *K*^+^ stimulus

**Fig 4:**
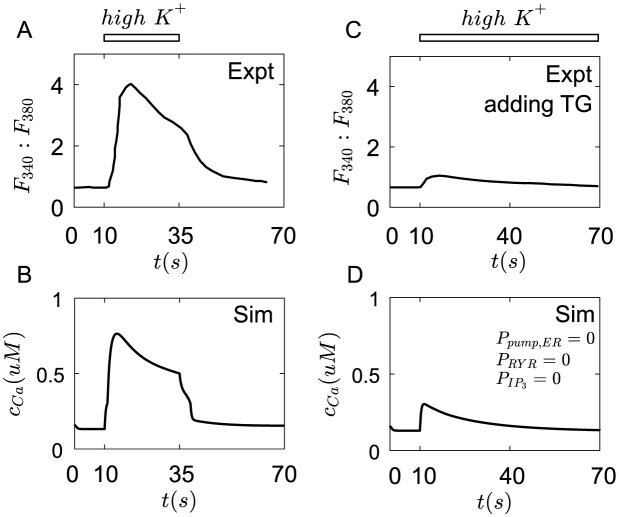
The *Ca*^2+^ transients in Merkel cells under high *K*^+^ stimulation. (A) The fluorescence intensity change of *Ca*^2+^ under high *K*^+^ solution [23]. (B) The cytoplasm concentration of *Ca*^2+^ change under high *K*^+^ solution: *c*_*K*,*out*_ increases by 130*nM* and *c*_*Na*,*out*_ reduces by 130*nM* in simulation. (C) The fluorescence intensity change of *Ca*^2+^ under high *K*^+^ solution by adding thapsigargin (TG), which is a specific blocker of *Ca*^2+^ pumps on ER, to deplete intracellular *Ca*^2+^ stores [23]. (D) The cytoplasm concentration of *Ca*^2+^ change under high *K*^+^ solution in simulation by setting *P*_*RYR*_ = 0, *P*_*leak*_ = 0, $P_{IP_{3}}=0$, and *P*_*pump*,*ER*_ = 0. The parameters of ion channels are seen in Table A in S1 Appendix.

High *K*^+^ solutions are made by reducing *Na*^+^ and increasing *K*^+^ concentrations in external solutions [22, 23]. The experimental results from [23] are shown in Fig 4A. Here we increase *C*_*K*,*out*_ by 130*mM*, and reduce *C*_*Na*,*out*_ by 130*mM* to represent the high *K*^+^ solution. The results show that the high *K*^+^ solution induces a rapid increase in *Ca*^2+^ concentration. After reaching a peak, *Ca*^2+^ concentration decreases slowly(Fig 4B). This *Ca*^2+^ transient could last for tens of seconds in Merkel cells, which is much longer than the timescale of membrane potential dynamics(Fig 2). This *Ca*^2+^ transient is regulated by both plasma membrane *Ca*^2+^ channels and internal *Ca*^2+^ channels in Merkel cells. The inhibition of ER *Ca*^2+^ stores causes a dramatic drop in fluorescence intensity of *Ca*^2+^(Fig 4C). By setting the rate constants of *Ca*^2+^ channels on ER to zero, we can simulate a similar dramatic drop of *Ca*^2+^ concentration(Fig 4D).

#### Hypotonic shock

**Fig 5:**
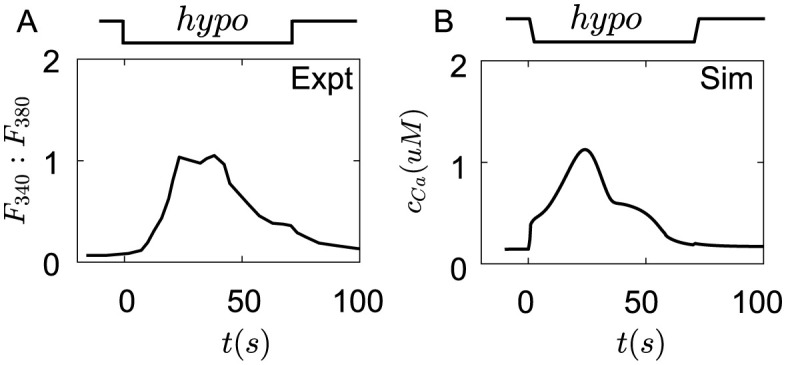
The *Ca*^2+^ transients in Merkel cells under hypotonic shock. (A) The fluorescence intensity change of *Ca*^2+^ under hypotonic shock by removing 30*mM* mannitol in external solution [22]. (B) The cytoplasm concentration of *Ca*^2+^ changes under hypotonic shock by removing 30*mM* mannitol in external solution in simulation. The parameters of ion channels are seen in Table A in S1 Appendix.

The hypotonic shock also results in *Ca*^2+^ transients in Merkel cells. Merkel cells were cultured in solutions that remove a part of *Na*^+^ and add the same concentration of mannitols. Then hypotonic shock was induced by removing mannitols in solutions [22, 23]. The hypotonic shock induces *Ca*^2+^ to enter the cell, and the fluorescence intensity of *Ca*^2+^ increase slowly, after reaching a peak, *Ca*^2+^ concentration goes back to the baseline(Fig 5A) [22]. In the simulation, hypotonic shock causes the opening of *Piezo*2 channels, *Ca*^2+^ concentration increases and reaches the peak, then gradually decreases(Fig 5B). But *Ca*^2+^ transients in the experiments of [22] increase slowly while the *Ca*^2+^ transients in the simulation have an immediate increase. An important reason is that hypotonic shock can inhibit the cytoplasmic substances’ mobility [70], which hinders the quick entry of *Ca*^2+^ into the cell and diffusion of *Ca*^2+^ in the cell. However, our model didn’t take this inhibitory role of hypotonic shock into account, which is a limitation of our model.

### Effects of ion channels properties on membrane potentials and *Ca*^2+^ transients

According to experiments from [21–24], Merkel cells in different locations of skin, different animals, or different experimental conditions have different expression of ions channels. For example, in experiments of yamashita [21], currents across *K*_*v*_1.4 and *K*_*v*_4.2 channels with inactivation property are dominated in total *K*^+^ currents. While in experiments of piskorowski [23], *BKCa* channels carry 50 ∼ 80% of total *K*^+^ currents. The currents only have a slight decrease with time. Therefore, it is reasonable that these channels have various conductances in different Merkel cells. The results from different experiments and our simulation results are consistent with the opinion that these ion channels and pumps in Merkel cells are the keys that regulate the dynamics of membrane potentials and *Ca*^2+^ transients. However, how these channels control the membrane potentials and *Ca*^2+^ transients is still unclear.

#### Membrane potentials

**Fig 6:**
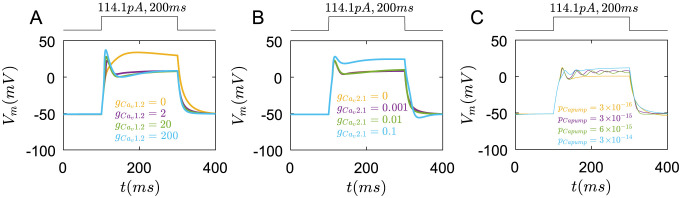
The roles of *Ca*_*v*_1.2, *Ca*_*v*_2.1 channels, and *Ca*^2+^ pumps in membrane potential regulation under the rectangular current pulse(114.1*pA*,200*ms*). (A) The changes of membrane potential with different $g_{Ca_{v}1.2}$, yellow line: $g_{Ca_{v}1.2}=0$, purple line: $g_{Ca_{v}1.2}=2$, green line: $g_{Ca_{v}1.2}=20$, blue line: $g_{Ca_{v}1.2}=200(mS/cm^{2})$. (B) The changes of membrane potential with different $g_{Ca_{v}2.1}$, yellow line: $g_{Ca_{v}2.1}=0$, purple line: $g_{Ca_{v}2.1}=0.001$, green line: $g_{Ca_{v}2.1}=0.01$, blue line: $g_{Ca_{v}2.1}=0.1(mS/cm^{2})$. (C) The changes of membrane potential with different *P*_*Capump*_. yellow line: *P*_*Capump*_ = 3 × 10^−16^, purple line: *P*_*Capump*_ = 3 × 10^−15^, green line: *P*_*Capump*_ = 6 × 10^−15^, blue line: *P*_*Capump*_ = 3 × 10^−14^(*mol*/(*cm*^2^ ⋅ *ms*)).

First, we study how *Ca*^2+^ channels regulate the membrane potentials of Merkel cells. By changing the specific membrane conductance of *Ca*_*v*_1.2 channels, we find that, when *g*_*Cav*1.2_ is zero, the membrane potential rapidly increases initially, then reaches a steady state gradually. There is no sharp peak for membrane potential(Fig 6A yellow line). With the increase of *g*_*Ca*_*v*_1.2_, the membrane potential rapidly rises to its peak. After a small drop, the membrane potential gradually stabilizes(Fig 6A purple line). The peak value of membrane potentials increases with $g_{Ca_{v}1.2}$(Fig 6A).

Compared to *Ca*_*v*_1.2 channels, *Ca*_*v*_2.1 channels have little role in the peak form of membrane potentials. When $g_{Ca_{v}2.1}$ is small, they almost do not affect the membrane potentials(Fig 6B). As $g_{Ca_{v}2.1}$ increases to 0.1, the membrane potential has a higher steady value(Fig 6B blue line).

However, the phenomenon of membrane potential fire(oscillation) does not happen whatever $g_{Ca_{v}1.2}$ or $g_{Ca_{v}2.1}$ changes. Interestingly, the oscillations of membrane potentials only appear when *p*_*Capump*_ is in the right range (Fig 6C purple and green line). When *p*_*Capump*_ is out of the range, the membrane potentials reach a steady state (Fig 6C yellow and blue line).

**Fig 7:**
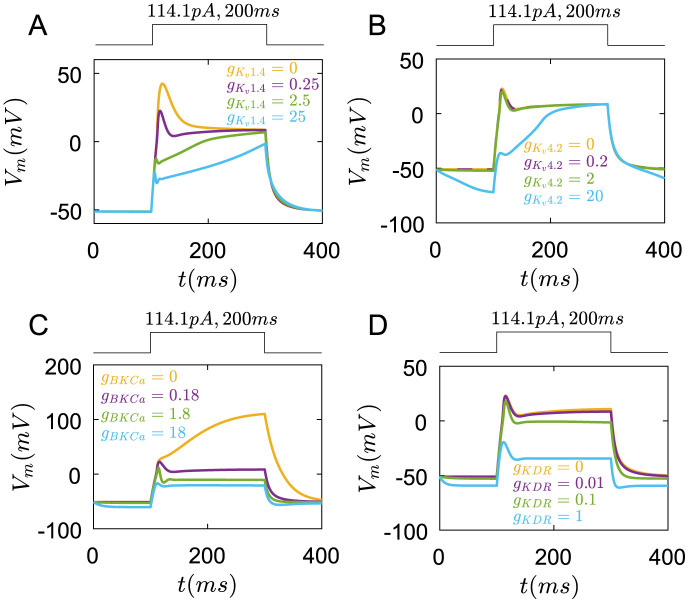
The roles of *K*_*v*_1.4, *K*_*v*_4.2, *BKCa*, and *KDR* channels in membrane potential regulation under the rectangular current pulse(114.1*pA*,200*ms*). (A) The changes of membrane potential with different $g_{K_{v}1.4}$. yellow line: $g_{K_{v}1.4}=0$, purple line: $g_{K_{v}1.4}=0.25$, green line: $g_{K_{v}1.4}=2.5$, blue line: $g_{K_{v}1.4}=25(mS/cm^{2})$. (B) The changes of membrane potential with different $g_{K_{v}4.2}$. yellow line: $g_{K_{v}4.2}=0$, purple line: $g_{K_{v}4.2}=0.2$, green line: $g_{K_{v}4.2}=2$, blue line: $g_{K_{v}4.2}=20(mS/cm^{2})$. (C) The changes of membrane potential with different *g*_*BKCa*_. yellow line: *g*_*BKCa*_ = 0, purple line: *g*_*BKCa*_ = 0.18, green line: *g*_*BKCa*_ = 1.8, blue line: *g*_*BKCa*_ = 18(*mS*/*cm*^2^). (D) The changes of membrane potential with different *g*_*KDR*_. yellow line: *g*_*KDR*_ = 0, purple line: *g*_*KDR*_ = 0.01, green line: *g*_*KDR*_ = 0.1, blue line: *g*_*KDR*_ = 1(*mS*/*cm*^2^).

Next, we study the roles of *K*^+^ channels on membrane potentials. As $g_{K_{v}1.4}$ is zero, the membrane potential has a bigger peak(Fig 7A yellow line). With the increase of $g_{K_{v}1.4}$, the peak value of membrane potentials decreases(Fig 7A). This result indicates that *K*_*v*_1.4 channels inhibit the peak of membrane potentials caused by *Ca*^2+^ channels.

As $g_{K_{v}4.2}$ is zero or small, they have little influence on membrane potentials(Fig 7B yellow line). This also means that *K*_*v*_1.4 channels mainly regulate the membrane potential peak in normal situations. As $g_{K_{v}4.2}$ increases to 20, *K*_*v*_4.2 channels greatly reduce the resting membrane potential, further suppressing the action potential peak(Fig 7B blue line).

When *g*_*BKCa*_ is zero, the membrane potential reaches the normal peak but continues to increase over 100*mV*(Fig 7C yellow line). As *g*_*BKCa*_ increases, the membrane potentials decrease to a steady state after reaching the peak(Fig 7C). The steady value of membrane potentials decreases with the increase of *g*_*BKCa*_(Fig 7C).

When *g*_*KDR*_ is zero, the membrane potentials have a little change(Fig 7D yellow line). This result also indicates that *BKCa* channels mainly control the steady membrane potentials. As *g*_*KDR*_ increases, the steady membrane potentials also reduce(Fig 7D).

In experiments of [21], the inhibition of *K*^+^ channels and the enhancement of *Ca*^2+^ channels by the external solution containing *Ba*^2+^ make Merkel cells depolarize at smaller currents injection. This is consistent with our results that with the decrease of conductances of *K*^+^ channels like *K*_*v*_1.4, *BKCa*, and *KDR* channels, the depolarized membrane potentials are more positive(Fig 7A, 7C and 7D). The membrane potentials also form the peak at smaller currents injection, which is consistent with the increase of $g_{Ca_{v}1.2}$ and the decrease of $g_{K_{v}1.4}$ cause the peak of membrane potentials in simulation (Figs 6A and 7A).

**Fig 8:**
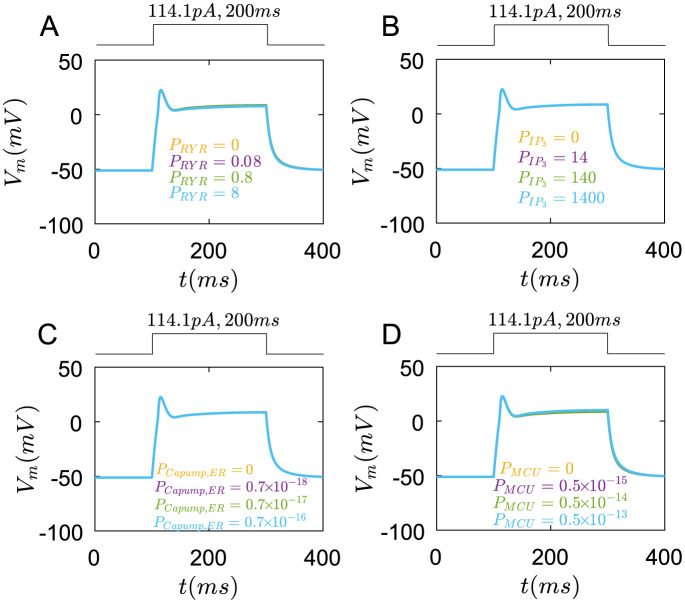
The roles of Ryanodine receptors, *IP*_3_ receptors, ER *Ca*^2+^ pumps and MCU pumps in membrane potential regulation under the rectangular current pulse(114.1*pA*,200*ms*). (A) The changes of membrane potential with different *P*_*RYR*_. yellow line: *P*_*RYR*_ = 0, purple line: *P*_*RYR*_ = 0.08, green line: *P*_*RYR*_ = 0.8, blue line: *P*_*RYR*_ = 8(*cm*/*ms*). (B) The changes of membrane potential with different $P_{IP_{3}}$. yellow line: $P_{IP_{3}}=0$, purple line: $P_{IP_{3}}=14$, green line: $P_{IP_{3}}=140$, blue line:$P_{IP_{3}}=1400$(*cm*/*ms*). (C) The changes of membrane potential with different *P*_*Capump*,*ER*_. yellow line: *P*_*Capump*,*ER*_ = 0, purple line: *P*_*Capump*,*ER*_ = 0.7 × 10^−18^, green line: *P*_*Capump*,*ER*_ = 0.7 × 10^−17^, blue line: *P*_*Capump*,*ER*_ = 0.7 × 10^−16^(*mol*/(*cm*^2^⋅*ms*)). (D) The changes of membrane potential with different *P*_*MCU*_(*P*_*MCU*_/*P*_*MNCX*_ = 5). yellow line: *P*_*MCU*_ = 0, purple line: *P*_*MCU*_ = 0.5 × 10^−15^, green line: *P*_*MCU*_ = 0.5 × 10^−14^, blue line: *P*_*MCU*_ = 0.5 × 10^−13^(*mol*/(*cm*^2^ ⋅ *ms*)).

Finally, we also study the influences of internal *Ca*^2+^ receptors and channels on membrane potentials. However, they almost have no function on membrane potentials(Fig 8).

Together, these results indicate that *Ca*_*v*_1.2 channels mainly help to form the peak of membrane potentials while *K*_*v*_1.4 channels directly reduce this peak. The coupling between *Ca*_*v*_2.1, *Ca*_*v*_1.2 channels, and *Ca*^2+^ pumps contribute to the oscillation of membrane potentials. *BKCa* and *K*_*KDR*_ channels are mainly to maintain a lower steady membrane potential.

#### Ca^2+^ transients

**Fig 9:**
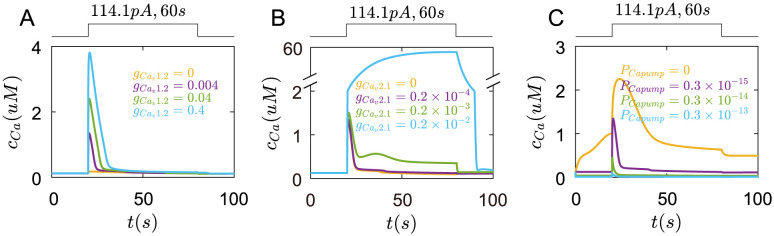
The roles of *Ca*_*v*_1.2, *Ca*_*v*_2.1 channels, and *Ca*^2+^ pumps in *Ca*^2+^ transients under the rectangular current pulse(114.1*pA*,60*s*). (A) The changes of *Ca*^2+^ concentration in the cell with different $g_{Ca_{v}1.2}$, yellow line: $g_{Ca_{v}1.2}=0$, purple line: $g_{Ca_{v}1.2}=0.004$, green line: $g_{Ca_{v}1.2}=0.04$, blue line: $g_{Ca_{v}1.2}=0.4(mS/cm^{2})$. (B) The changes of *Ca*^2+^ concentration in the cell with different $g_{Ca_{v}2.1}$, yellow line: $g_{Ca_{v}2.1}=0$, purple line: $g_{Ca_{v}2.1}=0.2\times 10^{-4}$, green line: $g_{Ca_{v}2.1}=0.2\times 10^{-3}$, blue line: $g_{Ca_{v}2.1}=0.2\times 10^{-2}(mS/cm^{2})$. (C) The changes of *Ca*^2+^ concentration in the cell with different *P*_*Capump*_. yellow line: *P*_*Capump*_ = 0, purple line: *P*_*Capump*_ = 0.3 × 10^−15^, green line: *P*_*Capump*_ = 0.3 × 10^−14^, blue line: *P*_*Capump*_ = 0.3 × 10^−13^(*mol*/(*cm*^2^ ⋅ *ms*)).

First, when $g_{Ca_{v}1.2}$ is zero, cytoplasmic *Ca*^2+^ concentration only increases slightly under current stimulation(Fig 9A yellow line). As $g_{Ca_{v}1.2}$ increases, cytoplasmic *Ca*^2+^ concentration has a rapid rise and fall (Fig 9A). The peak of *c*_*Ca*_ increases with $g_{Ca_{v}1.2}$.

When $g_{Ca_{v}2.1}$ is zero, they don’t affect *Ca*^2+^ transients(Fig 9B). As $g_{Ca_{v}1.2}$ increases, the *Ca*^2+^ concentration not only has a bigger peak but also keeps at a high level for a longer time(Fig 9B green line). However, if $g_{Ca_{v}2.1}$ increases to 0.002, the *Ca*^2+^ concentration rises over to 50*μM*(Fig 9B blue line), which less happens in normal condition. These results are consistent with the results that the inhibition of *Ca*_*v*_1.2 or *Ca*_*v*_2.1 reduces the *Ca*^2+^ transients in experiments of [12].

Oppositely, when *P*_*Capump*_ is zero, the resting *Ca*^2+^ concentration is high(Fig 9C yellow line), and the *Ca*^2+^ transients elicited by the current pulse are more obvious. As *P*_*Capump*_ increases, the resting *Ca*^2+^ concentration gets smaller, and *Ca*^2+^ transients are also weak(Fig 9C).

**Fig 10:**
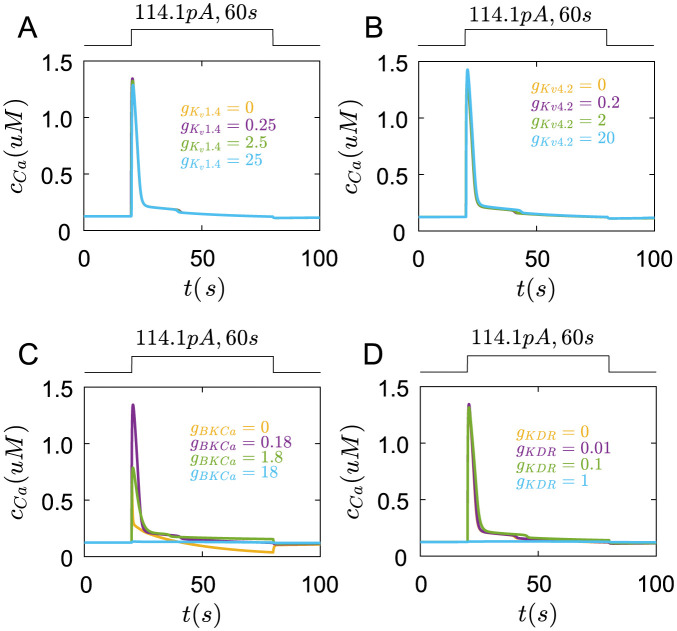
The roles of *K*_*v*_1.4, *K*_*v*_4.2, *BKCa* and *KDR* channels in *Ca*^2+^ transients under the rectangular current pulse(114.1*pA*,60*s*). (A) The changes of *Ca*^2+^ concentration in the cell with different $g_{K_{v}1.4}$. yellow line: $g_{K_{v}1.4}=0$, purple line: $g_{K_{v}1.4}=0.25$, green line: $g_{K_{v}1.4}=2.5$, blue line: $g_{K_{v}1.4}=25(mS/cm^{2})$. (B) The changes of *Ca*^2+^ concentration in the cell with different $g_{K_{v}4.2}$. yellow line: $g_{K_{v}4.2}=0$, purple line: $g_{K_{v}4.2}=0.2$, green line: $g_{K_{v}4.2}=2$, blue line: $g_{K_{v}4.2}=20(mS/cm^{2})$. (C) The changes of *Ca*^2+^ concentration in the cell with different *g*_*BKCa*_. yellow line: *g*_*BKCa*_ = 0, purple line: *g*_*BKCa*_ = 0.18, green line: *g*_*BKCa*_ = 1.8, blue line: *g*_*BKCa*_ = 18(*mS*/*cm*^2^). (D) The changes of *Ca*^2+^ concentration in the cell with different *g*_*KDR*_. yellow line: *g*_*KDR*_ = 0, purple line: *g*_*KDR*_ = 0.01, green line: *g*_*KDR*_ = 0.1, blue line: *g*_*KDR*_ = 1(*mS*/*cm*^2^).

Next, *K*_*v*_1.4 and *K*_*v*_4.2 channels both have a little role in *Ca*^2+^ transients(Fig 10A and 10B).

When *g*_*BKCa*_ is zero, the *Ca*^2+^ concentration rapidly rises to a peak, then enters a long recovery process(Fig 10C yellow line). As *g*_*BKCa*_ increases to 0.18, the *Ca*^2+^ cncentration reaches a bigger peak(Fig 10C purple line). But when *g*_*BKCa*_ increase to 1.8, the peak of *Ca*^2+^ concentration decreases. If *g*_*BKCa*_ is big enough, the *Ca*^2+^ transients are inhibited (Fig 10C green and blue line).

When *KDR* is zero or small, they have little role in the changes of *Ca*^2+^ concentration(Fig 10D). Only *g*_*KDR*_ is big enough, the *Ca*^2+^ concentration has a very slight increase(Fig 10D blue line).

**Fig 11:**
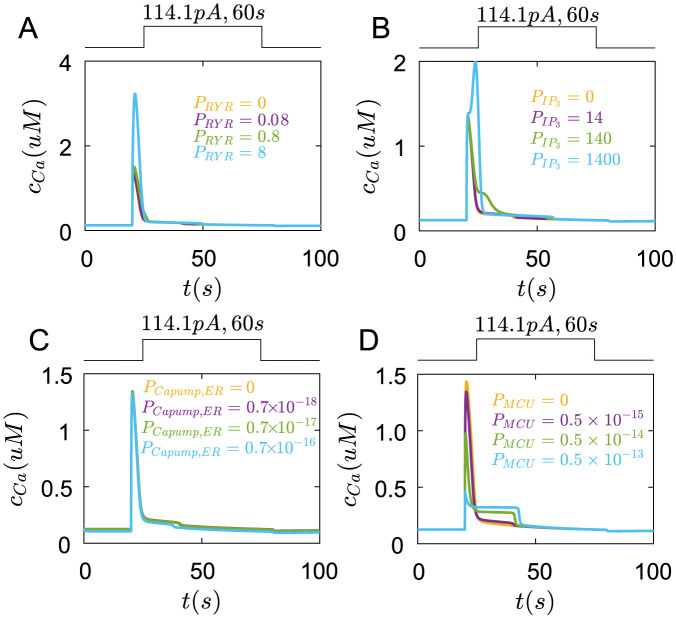
The roles of Ryanodine receptors, *IP*_3_ receptors, ER *Ca*^2+^ pumps and MCU pumps in *Ca*^2+^ transients under the rectangular current pulse(114.1*pA*,200*ms*). (A) The changes of *Ca*^2+^ concentration in the cell with different *P*_*RYR*_. yellow line: *P*_*RYR*_ = 0, purple line: *P*_*RYR*_ = 0.08, green line: *P*_*RYR*_ = 0.8, blue line: *P*_*RYR*_ = 8(*cm*/*ms*). (B) The changes of membrane potential with different $P_{IP_{3}}$. yellow line: $P_{IP_{3}}=0$, purple line: $P_{IP_{3}}=14$, green line: $P_{IP_{3}}=140$, blue line:$P_{IP_{3}}=1400$(*cm*/*ms*). (C) The changes of *Ca*^2+^ concentration in the cell with different *P*_*Capump*,*ER*_. yellow line: *P*_*Capump*,*ER*_ = 0, purple line: *P*_*Capump*,*ER*_ = 0.7 × 10^−18^, green line: *P*_*Capump*,*ER*_ = 0.7 × 10^−17^, blue line: *P*_*Capump*,*ER*_ = 0.7 × 10^−16^(*mol*/(*cm*^2^ ⋅ *ms*)). (D) The changes of *Ca*^2+^ concentration in the cell with different *P*_*MCU*_(*P*_*MCU*_/*P*_*MNCX*_ = 5). yellow line: *P*_*MCU*_ = 0, purple line: *P*_*MCU*_ = 0.5 × 10^−15^, green line: *P*_*MCU*_ = 0.5 × 10^−14^, blue line: *P*_*MCU*_ = 0.5 × 10^−13^(*mol*/(*cm*^2^ ⋅ *ms*)).

Finally, the internal *Ca*^2+^ stores have an obvious impact on *Ca*^2+^ transients. As *P*_*RYR*_ is small, the *Ca*^2+^ transients are almostly unchanged(Fig 11A yellow line). When *P*_*RYR*_ is big, the *Ca*^2+^ concentration rises to a bigger peak(Fig 11A blue line).

When $P_{IP_{3}}$ is small, they also do not alter the *Ca*^2+^ transients(Fig 11B yellow and purple line). This indicates that the *Ca*^2+^ concentration increase at the beginning of the current stimulus is mostly controlled by *Ca*_*v*_1.2 and *Ca*_*v*_2.1. As $P_{IP_{3}}$ increases to 140, the *Ca*^2+^ transients sustain for a longer time(Fig 11B green line). If $P_{IP_{3}}$ is big enough, the *Ca*2+ concentration has a bigger peak(Fig 11B blue line).

The ER *Ca*^2+^ pumps have a slight role in *Ca*^2+^ transients(Fig 11C).

When *P*_*MCU*_ is zero, the peak of *Ca*^2+^ concentration is bigger than the normal situation(Fig 11D yellow line). As *P*_*MCU*_ increases, the peak of *Ca*^2+^ concentration decreases but the duration of *Ca*^2+^ transients increases(Fig 11D).

Together, these results indicate that *Ca*_*v*_1.2 and *Ca*_*v*_2.1 channels, Ryanodine and *IP*_3_ receptors could increase the *Ca*^2+^ transients, while *BKCa* and *KDR* channels reduce the *Ca*^2+^ transients. Combining the inhibition roles of *BKCa* and *KDR* on membrane potentials, it can be speculated that *BKCa* and *KDR* channels, by controlling the resting membrane potentials, further regulate the voltage-gated *Ca*^2+^ channels to influence the *Ca*^2+^ transients. *K*_*v*_1.4 and *K*_*v*_4.2 channels only influence membrane potentials at the initial time, which have less role in *Ca*^2+^ transients. In turn, the depolarization of membrane potentials facilitates the *Ca*^2+^ channels also regulate membrane potentials. Therefore, the membrane potentials and the *Ca*^2+^ transients in Merkel cells are coupled to each other.

### How Merkel cells respond to mechanical stimulus

We have studied how ion channels on Merkel cells shape the cell membrane potentials and *Ca*^2+^ transients. However, Merkel cells are mechanical sensory cells, which could transduce tactile stimuli to SA1 afferents [6, 8, 24]. *Piezo*2 channels are necessary for this transduction [7, 9]. The SA1 afferents generate continuous action potentials under a static mechanical displacement. Without Merkel cells, SA1 afferents only generate action potentials at the initial moment of stimulation. The knockdown of *Piezo*2 channels in the Mekel cells causes similar results. A recent study held the view that *Piezo*2 channels in Merkel cells are not enough to help SA1 afferents generate continuous action potentials [11]. Therefore, we want to know how *Piezo*2 channels and other ion channels in Merkel cells participate in this process. The model is modified as follows.

#### Exocytosis and endocytosis

Merkel cells transmit information to downstream afferents by releasing neurotransmitters [15, 16]. Generally, the synapse release can be divided into three parts. First, cell organelles synthesize neurotransmitters into vesicles. Many vesicles form the vesicles pools in the presynapse [71, 72]. Second, When *Ca*^2+^ ions enter the cell or cytoplasm *Ca*^2+^ concentration increases, vesicles in the pools fuse to the cell membrane(exocytosis) and release neurotransmitters [15, 16]. Finally, vesicles fused to the membrane recycle into the cell by endocytosis [73, 74]. Vesicles in the pools keep a relatively fixed number at rest, and the consumption of vesicles can be rapidly replenished. Then we assume that the vesicle synthesis rate is inversely related to the number of vesicles. The exocytosis rate increases with the cytoplasm *Ca*^2+^ concentration [20], and is positively correlated with the number of vesicles. Thus the dynamic equation of vesicles can be written as

$$\begin{array}{c} \frac{dn_{ve}}{dt}=\frac{k_{ve}}{1+exp(-\frac{n_{ve}-n_{ve,s}}{n_{ve,f}})}-\frac{k_{exo}}{1+exp(-\frac{c_{Ca}-c_{Ca,s}}{c_{Ca,f}})}n_{ve}, \end{array}$$ (86)

where *n*_*ve*_ is the number of vesicles, *k*_*ve*_ and *k*_*exo*_ are the rate constants. *n*_*ve*,*s*_, *n*_*ve*,*f*_, *c*_*Ca*,*s*_, and *c*_*Ca*,*f*_ are constants.

Endocytosis is regulated by many complex signals, one of which is membrane tension [75–77]. Generally, when the membrane tension is great, more energy is required for vesicles to form from the cell membrane. Therefore, the endocytosis rate can take the form of $\frac{k_{endo}}{1+exp(\frac{\sigma -\sigma_{s,ve}}{\sigma_{f,ve}})}$ [77, 78], where *k*_*endo*_ is rate constant. *σ*_*s*,*ve*_ and *σ*_*f*,*ve*_ are constants. Endocytosis reduces the cell membrane, while exocytosis adds the cell membrane. Thus the reference surface *S*_*ref*_ changes as

$$\begin{array}{c} \frac{dS_{ref}}{dt}=4\pi r_{ve}^{2}\left(\frac{k_{exo}}{1+exp(-\frac{c_{Ca}-c_{Ca,s}}{c_{Ca,f}})}n_{ve}-\frac{k_{endo}}{1+exp(\frac{\sigma -\sigma_{s,ve}}{\sigma_{f,ve}})}\right), \end{array}$$ (87)

where *r*_*ve*_ is the average radius of vesicles. The values of parameters are seen in Table 4.

**Table 4:** Parameters of endocytosis and exocytosis.

Parameter	Description	Value in simulation
*k*_*ve*_	Vesicle forming rate (1/*ms*)	0.1 [79]
*n*_*ve*,*s*_	Reference vesicles number (1)	500 [66, 80]
*n*_*ve*,*f*_	Reference vesicles number (1)	50 [66, 80]
*k*_*exo*_	Exocytosis rate constant (1/*ms*)	2 × 10^−4^ [75, 81]
*c*_*Ca*,*s*_	Reference *Ca*^2+^ concentration (*μM*)	0.2 [82]
*c*_*Ca*,*f*_	Reference *Ca*^2+^ concentration (*μM*)	0.01 [82]
*r*_*ve*_	Vesicles radius (*μm*)	0.05 [66]
*k*_*endo*_	Endocytosis rate constant (1/*ms*)	1 [75, 78, 80]
*σ*_*ve*,*s*_	Reference cortex stress (*Pa*)	1000 [78]
*σ*_*ve*,*f*_	Reference cortex stress (*Pa*)	15 [78]

#### Cell compression

The Merkel cell is a sphere before indentation, it is compressed to a cylinder of changeable radius *r* as shown in Fig A in S1 Appendix. It’s height *H* decreases with compression depth *d*,

$$\begin{array}{c} H=2r_{ini}-d, \end{array}$$ (88)

where *r*_*ini*_ is the initial cell radius before compression. The other equations about the cell deformation are seen in Cell indentation in S1 Appendix.

**Fig 12:**
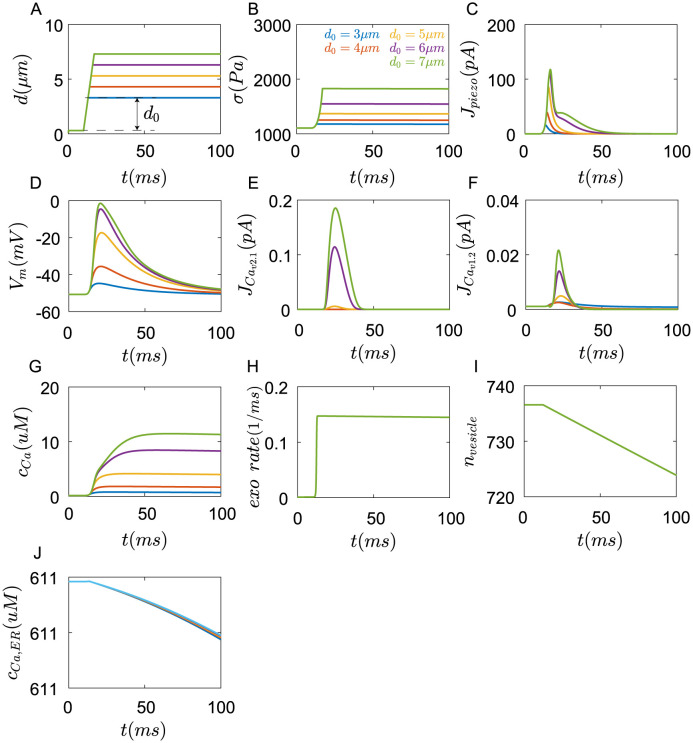
Responses of Merkel cells under compression in 100*ms*. (A) The Merkel cell was compressed at a depth *d*_0_ with a speed of 1*um*/*ms*. The dynamic change of (B) cortex stress, (C) currents generated in the *Piezo*2 channels, (D) membrane potential, (E) currents generated in *Ca*_*v*_2.1 channels, (F) currents generated in *Ca*_*v*_1.2 channels, (G) cytoplasmic concentration of *Ca*^2+^, (H) exocytosis rate, (I) number of cytoplasmic vesicles, and (J) Endoplasmic reticulum concentration of *Ca*^2+^(blue:*d*_0_ = 3*μm*, red:*d*_0_ = 4*μm*, yellow:*d*_0_ = 5*μm*, cyan:*d*_0_ = 6*μm*, green:*d*_0_ = 7*μm*).

The results show that when the Merkel cell was compressed(Fig 12A), the shape change of the cell causes the increase of cortex stress *σ* (Fig 12B), further activates *Piezo*2 channels(Fig 12C), *Ca*^2+^ flow through *Piezo*2 channels into the cell, which causes the depolarization of membrane potential(Fig 12D). The increase of membrane potential opens *Ca*_*v*_2.1 and *Ca*_*v*_1.2 channels(Fig 12E and 12F). The *Ca*^2+^ flow across *Piezo*2, *Ca*_*v*_2.1, and *Ca*_*v*_1.2 channels results in the increase of *Ca*^2+^ concentration(Fig 12G), further promoting exocytosis (Fig 12H). Then vesicles in the cell decrease (Fig 12I). Cytoplasmic *Ca*^2+^ further cause the *Ca*^2+^ release in the ER(Fig 12J).

*Piezo*2 channels inactivate rapidly under compression(Fig 12C). Without currents of *Piezo*2 channels, the membrane potentials repolarize(Fig 12H). It is consistent with the results of [7]. Voltage-gated *Ca*_*v*_2.1 and *Ca*_*v*_1.2 channels also close(Fig 12H). The *Ca*^2+^ concentration starts to decrease slowly(Fig 12G). With the increase of compression depth *d*_0_, the depolarization of membrane potential is enhanced, and the rise of *Ca*^2+^ concentration strengthens.

**Fig 13:**
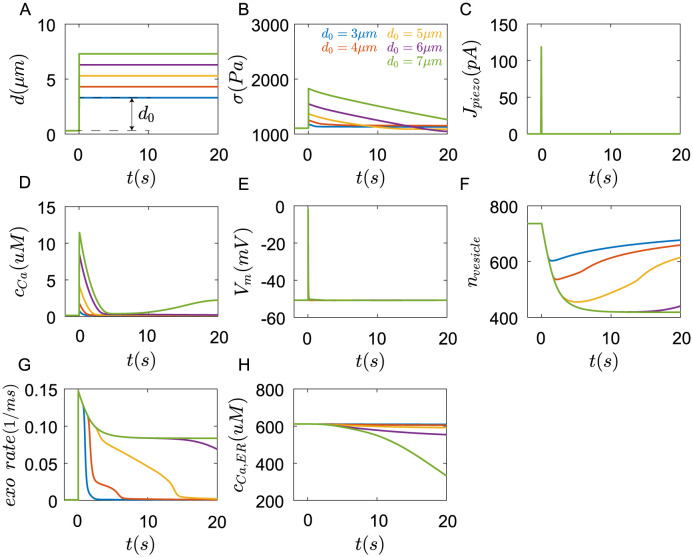
Responses of Merkel cells under compression in 20*s*. (A) The Merkel cell was compressed at a depth *d*_0_ with a speed of 1*um*/*ms*. The dynamic change of (B) cortex stress, (C) currents generated in the *Piezo*2 channels, (D) membrane potential, (E) cytoplasmic concentration of *Ca*^2+^, (F) number of cytoplasmic vesicles, (G) exocytosis rate, and (H) Endoplasmic reticulum concentration of *Ca*^2+^(blue:*d*_0_ = 3*μm*, red:*d*_0_ = 4*μm*, yellow:*d*_0_ = 5*μm*, cyan:*d*_0_ = 6*μm*, green:*d*_0_ = 7*μm*).

The above process happens in 100*ms*. After that, the concentration of *Ca*^2+^ continue to fall(Fig 13D), but the internal *Ca*^2+^ stores start to work. The *Ca*^2+^ in the ER enters the cell through Ryanodine and *IP*_3_ receptors, keeping a relatively high *Ca*^2+^ concentration, and continuously facilitating exocytosis to release neurotransmitters. Exocyotosis can last for tens of seconds. If we assume that neurotransmitters in every vesicle are close. Then the downstream afferents will receive continuous and stable neurotransmitter stimuli, which could generate continuous action potentials. The time of exocytosis increases with the compression depth, which is consistent with the results that the firing time of SAI Afferents increases with the indentation depth in experiments of [6, 7]. However, if *Piezo*2 channels were inhibited, no *Ca*^2+^ currents flow into the cell, and the membrane potential is at rest, Merkel cells will not respond to mechanical stimuli(Fig G in S1 Appendix), which is consistent with the results that the knockout of Piezo2 channel or inhibitory of Piezo2 channels by *Cd*^2+^ result in the loss of sustained action potentials in the SAI afferents [7, 24].

## Discussion

In this article, we develop a biophysically detailed model of Merkel cells for the reception of Merkel cells. We first validate our model of Merkel cells with previous experiments. We then discuss how these ion channels control the membrane potentials and *Ca*^2+^ transients in Merkel cells. Finally, we study how Merkel cells convert the mechanical stimuli into the release of neurotransmitters with the participation of *Piezo*2 channels, membrane potentials, and *Ca*^2+^ transients.

### The responses of Merkel cells under different stimuli

First, the membrane potentials of Merkel cells are controlled by *K*^+^ and *Ca*^2+^ channels. As shown in Fig 2, Merkel cells exhibit a nearly passive response under small current stimuli. As the injection currents increase, the membrane potential forms a peak and then has a small decrease to the steady state. How do *K*^+^ and *Ca*^2+^ contribute to this phenomenon? As the conductances of *K*_*V*_1.4, *BKCa*, and *KDR* decrease, the depolarized membrane potential increases, which is consistent with the results that Merkel cells are easier to depolarize with the inhibition of *K*^+^ channels [21]. Oppositely, the increase of the conductances of *Ca*^2+^ channels increases the peak of membrane potentials, which is also similar to the results that the membrane potentials form a peak with the small currents stimulus when *Ca*^2+^ channels were enhanced [21]. *Ca*^2+^ also can contribute to the oscillation of membrane potentials. As shown in Fig 3, The membrane potentials of Merkel cells initially have a rapid depolarization, then gradually enter an oscillation. By reducing the *Ca*^2+^ concentration in the external solution, this oscillation disappears [24]. In simulation, by changing the conductance of *Ca*^2+^ pumps, there is also the oscillation of membrane potentials. Though there are different *Ca*^2+^-relative membrane potentials behaviors in Merkel cells. However, it seems to have no obvious qualitative influence on the mechanical transduction of Merkel cells. The action potentials of downstream SAI afferents with two kinds of membrane potentials are still similar [7, 24]. There may be other roles for the oscillation of membrane potentials of Merkel cells, which needs further study. On the other hand, the oscillation of membrane potentials of Merkel cells is smaller than the amplitude of action potentials of neural cells. The membrane potentials are still limited on low depolarization state by *K*^+^ channels.

Secondly, a high *K*^+^ solution is a general way to stimulate Merkel cells, for the main *K*^+^ channels expressed in the cell membrane. High *K*^+^ solutions alter the Nerst potentials of *K*^+^, which causes the flow of *K*^+^ into the cell, and the membrane depolarizes. The *Ca*^2+^ channels were opened, and *Ca*^2+^ enter the cell. *Ca*^2+^ transients were inspired. This process both link with membrane *Ca*^2+^ channels and internal *Ca*^2+^ stores. The inhibition of internal *Ca*^2+^ stores greatly reduce the amplitude of *Ca*^2+^ transients(Fig 4), which is consistent with the results in [23]. The decrease of the conductances of *Ca*_*v*_1.2 and *Ca*_*v*_2.1 channels also reduces the *Ca*^2+^ transients(Fig 9), with is consistent with that the inhibition of *Ca*_*v*_1.2 and *Ca*_*v*_2.1 reduces the *Ca*^2+^ transients in experiments of [12].

Finally, the hypotonic shock also causes the *Ca*^2+^ transients(Fig 5), different from the previous two stimuli, hypotonic shock is more like a mechanical stimulus. Piezo2 channels were opened with the increase of cortex stress for the water absorption of the cell. The membrane potential increases, and *Ca*^2+^ enters the cell. It can be seen that the differences of *Ca*^2+^ transients between experiments [22] and simulation(Fig 5). The *Ca*^2+^ concentration in the simulation almost immediately increases, then gradually rises to the peak. However, in the experiments of [22, 23], the *Ca*^2+^ transients at the beginning of stimulation increase slowly, then rise and fall. One of the possible reasons is that hypotonic shock can inhibit the cytoplasmic substances’ mobility [70], thus hindering the quick entry of *Ca*^2+^ into the cell and the diffusion of *Ca*^2+^ in the cell. However, this role of hypotonic shock has not been confirmed, which is a limitation of our model.

### How Merkel cells with Piezo2 channels help *Aβ* afferents generate sustained action potentials

According to our model, when Merkel cells are compressed, the opening of *Piezo*2 channels leads to the depolarization of membrane potentials and the influx of *Ca*^2+^. The increase of membrane potential further causes the influx of *Ca*^2+^ through *Ca*_*v*_1.2 and *Ca*_*v*_2.1. *Ca*^2+^ in the cytoplasm causes the exocytosis of neurotransmitters. Due to the rapid inactivation of Piezo2 channels, the membrane potential goes back to the resting state within hundreds of milliseconds. The *Ca*^2+^ channels are also closed. The concentration of *Ca*^2+^ in the cytoplasm decreases. But the internal *Ca*^2+^ store begins to release *Ca*^2+^ into the cell, which can last for tens of seconds. Exocytosis lasts for the same amount of time until *Ca*^2+^ transients disappear. Therefore, Piezo2 channels excite Merkel cells to produce a sustained neurotransmitter release, which further helps *Aβ* generate prolonged action potentials. So why does the theoretical study of [11] have such a conclusion that RA MS channels like *Piezo*2 channels cannot account for the sustained responses of Merkel cell-neurite complexes? Because they treat Merkel cells as ordinary nerve cells. A typical feature of these kinds of cells like afferents is that they generate *Na*^+^-related action potentials. For typical nerve cells, an action potential travels through axons and arrives at the presynapse, which depolarizes the presynaptic membrane potential. Then voltage-gated *Ca*^2+^ channels on the presynaptic membrane are opened, allowing *Ca*^2+^ to enter the cell and activate neurotransmitters release [83, 84]. But the *Ca*^2+^ transients in these nerve cells quickly return to the baseline [32, 85]. The number of vesicles released is positively correlated with the number of arriving action potentials. Therefore, the action potentials of downstream nerve cells are also positively related to the action potentials of presynapse [86–88]. If the nerve cell is compressed, the opening of *Piezo*2 channels will only make one or several action potentials [46]. Therefore, the misuse of neural properties in Merkel cells leads to the wrong conclusion [11].

### Limitations of the model

Merkel cell-neurite complexes generate sustained action potentials with irregular intervals when a sustained mechanical stimulus is applied to the skin [3, 6, 7, 24]. This irregular interval of action potentials is also one of the important features of the SA1 response. At least in our results, the neurotransmitter release of one Merkel cell is regular. There are at least two aspects that may lead to this phenomenon. First, not only *Ca*^2+^, *IP*_3_, but also many other secondary messengers like ATP and cAMP, play important roles in exocytosis in many neurons [89–92]. These secondary messengers interact with each other, regulating the irregular exocytosis, which further contributes to the irregular intervals. Secondly, an afferent is usually linked with several Merkel cells [8, 93, 94]. It means that even if a single Merkel cell releases vesicles regularly, the combination of several or dozens of Merkel cells with small differences in channel properties may lead to irregularities in the whole action potentials. The research of [93] found that the different locations in the skin, cell size, and so on of Merkel cells may determine this irregular interval. Modeling virtual Merkel cells by different membrane capacitance, and different conductance of ion channels also helps downstream afferents generate irregular action potentials. Therefore, further studies on this irregular action potential are needed.

In this article, we didn’t consider the diffusion of *Ca*^2+^ and *IP*_3_ in the cytoplasm due to the rather small size of Merkel cells. However, the diffusion of *Ca*^2+^ may cause the *Ca*^2+^ wave within the cell. This means that the *Ca*^2+^ concentration in the cytoplasm will be nonuniform, which may lead to more complex *Ca*^2+^ behaviors. In previous studies, the *Ca*^2+^ transients in the same cell by two same stimuli can be different [22, 23].

Although the *Piezo*2 channels prefer *Ca*^2+^, *Ca*^2+^ in external solutions is much smaller than *Na*^+^ and *K*^+^. These two kinds of cation channels also flux through *Piezo*2 channels. Here we ignore ions other than *Ca*^2+^ in *Piezo*2 channels. It may have quantitative impacts on our results.

Generally speaking, neurotransmitters in vesicles are adequate. But for Merkel cells, the release of neurotransmitters lasts for tens of seconds, and the supplements of vesicles and neurotransmitters may need to be considered. Upon stimulation, the neurotransmitters released in the synapse and vesicles fused to the membrane will be absorbed back into the cell, and some of the neurotransmitters will also diffuse into adjacent solutions [75, 95, 96]. The time scales of reabsorption of neurotransmitters and vesicles may be out of sync. There is even a “kiss and run” approach to neurotransmitter release, where neurotransmitters are released into the synaptic cleft but the membrane of vesicles goes back to the cell immediately. Therefore, the recycling of neurotransmitters and vesicles may also influence the sustained response of Merkel cells to stimulation.

## Conclusion

Although it has been identified that Merkel cells transduce tactile stimuli to SA1 afferents by *Piezo*2 channels, it remains unclear how *Piezo*2 channels with fast-inactivation properties help Merkel cells and SA1 afferents generate a slowly adapting response to mechanical stimuli. Here, we develop a biophysically detailed model for the reception of Merkel cells.

We first validate our model with several experimental results [21–24]. Merkel cells exhibit an almost passive response to negative current and small positive current pulses. As the positive current increases, the membrane potential rises to a peak value and then gradually reaches a steady state. Merkel cells also exhibit *Ca*^2+^ transients lasting tens of seconds under high *K*^+^ solutions and hypotonic shock.

We then discuss how these ion channels control the membrane potentials and *Ca*^2+^ transients in Merkel cells. Our works show that *Ca*_*v*_1.2 channels contribute to the formation of the peak of membrane potentials, while *K*_*v*_1.4 channels reduce this peak. *Ca*_*v*_2.1, *BKCa*, and *KDR* channels mainly maintain the steady membrane potentials, *Ca*_*v*_2.1 channels increase the steady membrane potentials, and *BKCa* and *KDR* channels reduce the steady membrane potentials. Interestingly, the oscillations of membrane potentials require the coupling of *Ca*^2+^ channels and *Ca*^2+^ pumps. Compared to these channels on the membrane, the Ryanodine and *IP*_3_ receptors on ER have little role in membrane potentials. Our works also show that *Ca*_*v*_1.2 channels increase the peak concentrations of *Ca*^2+^, while *Ca*_*v*_2.1 channels mainly increase the duration of high *Ca*^2+^ concentrations. Both *BKCa* and *KDR* channels suppress the *Ca*^2+^ transients. It can be speculated that these two channels inhibit the depolarization of membrane potentials, thereby inhibiting the voltage-gated *Ca*^2+^ channels on the membrane. Additionally, both Ryanodine and *IP*_3_ receptors on ER increase the *Ca*^2+^ transients.

Finally, we show that *Piezo*2 channels and internal *Ca*^2+^ stores are sufficient to activate continuous neurotransmitter release to downstream *Aβ* afferents. Surprisingly, the membrane potentials seem not necessary for Merkel cells to transduce mechanical stimuli to afferents. The depolarization of membrane potentials caused by *Piezo*2 channels will rapidly repolarize for the inactivation of *Piezo*2 channels. Oppositely, *Ca*^2+^ flows into the cell through *Piezo*2 channels, which activates Ryanodine and *IP*_3_ receptors on ER to keep a continuous high *Ca*^2+^ concentration. This *Ca*^2+^ transients facilitate the release of neurotransmitters, and the duration of exocytosis is corresponding to the time of *Aβ* afferents firing [6, 7, 24].

Thus, unlike sensory cells that generate *Na*^+^-related action potentials, Merkel cells, through stable membrane potentials and *Ca*^2+^ transients regulation, maintain a relatively stable release of neurotransmitters to mechanical stimuli.

However, knowledge about the neurotransmitter interaction between Merkel cells and *Aβ* afferents is still lacking [97]. In further research, we hope to establish a complete model of the Merkel cell and *Aβ* afferents, which may provide a better description or understanding of the sensing process of the Merkel cell-neurite complexes.

## Supporting information

S1 Appendix Description of cell indentation and additional informations about this work. Fig A. The schematic diagram of Merkel cell under indentation. Fig B. *K*_*v*_1.4 channel. Fig C. *K*_*v*_4.2 channel. Fig D. *KDR* channel. Fig E. *Piezo*2 channel. Fig F. Merkel cell reaches a balanced state given an initial value at rest. Fig G. The responses of Merkel cells under compression. Fig H. The responses of the Merkel cell under three kinds of stimuli with the same parameters. Fig I. The influences of exocytosis rate on vesicle regulation. Table A. Ion channel parameters in high *K*^+^ solutions and hypotonic shock. Table B. Ion concentrations of external solutions. Table C. Initial values of variables.
(PDF)

S1 Data Code data of the model in this work. (ZIP)
