# Supplementary material for: How Merkel cells transduce mechanical stimuli: A biophysical model of Merkel cells
Source: PLoS Comput Biol. 2023 Dec 20;19(12):e1011720. doi: 10.1371/journal.pcbi.1011720 (PMC10732429; doi:10.1371/journal.pcbi.1011720)
Supplement: S1 Appendix — Fig A. The schematic diagram of Merkel cell under indentation. Fig B. Kv1.4 channel. Fig C. Kv4.2 channel. Fig D. KDR channel. Fig E. Piezo2 channel. Fig F. Merkel cell reaches a balanced state given an initial value at rest. Fig G. The responses of Merkel cells under compression. Fig H. The responses of the Merkel cell under three kinds of stimuli with the same parameters. Fig I. The influences of exocytosis rate on vesicle regulation. Table A. Ion channel parameters in high K+ solutions and hypotonic shock. Table B. Ion concentrations of external solutions. Table C. Initial values of variables. (PDF) [file pcbi.1011720.s001.pdf]

Supplementing Information: S1 Appendix.
How Merkel cells transduce mechanical stimuli: A biophysical model of Merkel cells
Fangtao Mao, Wenzhen Yang

Cell indentation.

We assume that the Merkel cell is a sphere before indentation, then it will be compressed to a cylinder of changeable radius r as shown in Fig A. It's height H decreases with indentation depth d,

$$H = 2r_{ini} - d, \tag{1}$$

where r_{ini} is the initial cell radius before compression. At this moment, for any section plane perpendicular to Z axis [1],

$$\Delta P \pi r^2 - F_c = 2\pi r h_c \sigma sin\theta, \tag{2}$$

where ΔP is the hydrostatic pressure difference across the membrane, F_c is the force loaded on the cell by the object, h_c is the thickness of the cortex, σ is the cortex stress, $tan\theta = dz/dr$. At the boundary, $\theta = 0$, $r = a$, where a is the contact radius between object and cell (Fig A). So $F_c = \Delta P \pi a^2$. Thus

$$r(\theta) = a\left[\sqrt{1 + (\frac{\sigma h_c}{\Delta P a})^2 \sin^2\theta} + \frac{\sigma h_c}{\Delta P a}\sin\theta\right]. \tag{3}$$

According to $dz/dr = \tan\theta$, integrating z over θ, then

$$z = a\left[E_1(\theta, -B^2) - E_2(\theta, -B^2)\right] - aB\cos\theta + C, \tag{4}$$

where $B = \sigma h_c/\Delta P a$, $E_1(\theta, m) = \int_0^\theta \sqrt{1 - m\sin^2\varphi}d\varphi$, $E_2(\theta, m) = \int_0^\theta 1/\sqrt{1 - m\sin^2\varphi}d\varphi$. C is constant. The boundary conditions are

$$z = 0 \qquad (\theta = 0), \tag{5}$$

$$z = \frac{2r_{ini} - d}{2} \qquad (\theta = \frac{\pi}{2}). \tag{6}$$

From the boundary conditions, we solve equation 4,

$$\frac{2r_{ini} - d}{2} = a\left[E_1(\frac{\pi}{2}, -B^2) - E_2(\frac{\pi}{2}, -B^2)\right] + aB. \tag{7}$$

With r and z, we can describe the cell shape. By integration, the cell lateral area S_{lat} and cell volume V are

$$S_{lat} = 4\pi(aB)^2\left[\frac{2}{B}E_1(\frac{\pi}{2}, -B^2) - \frac{1}{B}E_2(\frac{\pi}{2}, -B^2) + 2\right], \tag{8}$$

$$V = \frac{2\pi}{3}(aB)^3[\frac{1}{B}(\frac{1}{B^2} + 8)E_1(\frac{\pi}{2}, -B^2) - \frac{1}{B}(\frac{1}{B^2} + 4)E_2(\frac{\pi}{2}, -B^2) + (\frac{3}{B^2} + 8)]. \tag{9}$$

Parameters estimation.

Table 1: Piezo2 channels on the Merkel cell membrane are in the closed state (C) at rest. When the cell is compressed or stimulated in other ways, the cortex stress increases, Piezo2 channels transform from the closed state to the open state(O). With the increase of the cortex stress, the opening of Piezo2 channels also increases, or channels under closed state are less. Therefore, we assume that C_∞ decreases with the cortex stress. When the biggest currents appear, all channels get out from the closed state. Thus we get the relationship between cortex stress σ and C_∞ as shown in Fig EA. The time constants of C are the time constants of the rising section of currents in experiments of coste[2](Fig EB). The channels under open state quickly enter a short-time inactivation state(In). Then the time constant of the open state will be the time constant of the descending section of currents[2](Fig EC). After a short time, the currents of channels reach zero. This means that the steady short-time inactivation In_∞ should equal to $1 - C_\infty$. We also assume the time constants of short-time inactivation τ_{In} is the same with τ_C. The long-time inactivation of Piezo2 channels was found in another study of lewis[3]. But the relationship between h_{slow} and σ lacks. However, the features of Piezo2 channels are similar to features of fast inactivation MS channels in the experiments

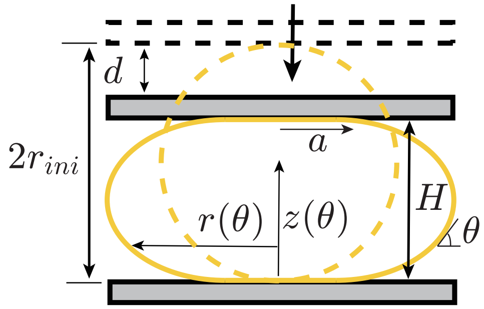

Fig A. The schematic diagram of Merkel cell under indentation.

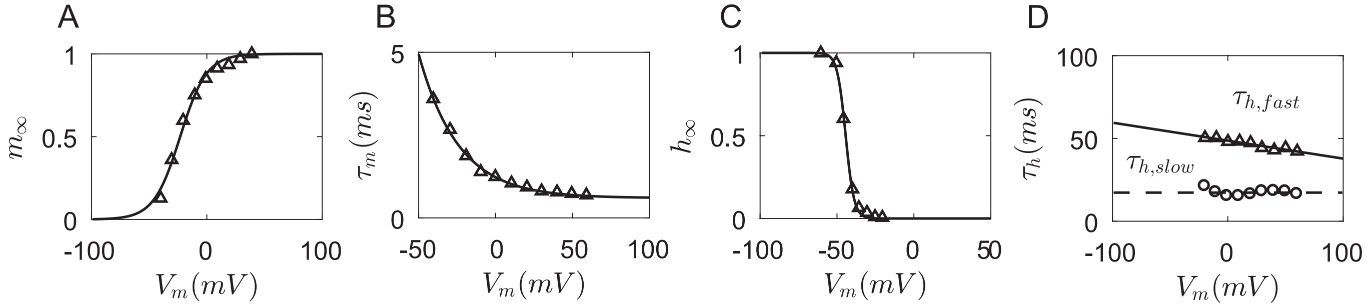

Fig B. $K_v1.4$ channel. (A) Voltage dependence of steady-state activation m_∞ (solid line), triangles represent the experimental data from [5] ($R^2 > 0.98$). (B) Voltage dependence of the activation time constant τ_m (solid line), triangles represent the experimental data from [5] ($R^2 > 0.99$). (C) Voltage dependence of steady-state inactivation h_∞ (solid line), triangles represent the experimental data from [5] ($R^2 > 0.99$). (D) Voltage dependence of the inactivation time constant $\tau_{h,fast}$ (solid line), triangles represent the experimental data from [5] ($R^2 > 0.91$),$\tau_{h,slow}$ (circle) seems voltage-independent, here we take the mean of data.

of delmas[4]. Therefore we adopt the parameters of long-time inactivation of the fast inactivation MS channels from delmas[4].

Table 2: The dissociation constant K_{ERpump} was adopted from the study of Fink[7]. The IP_3 receptors are activated by internal Ca^{2+} and IP_3. Their maximum currents present at $C_{Ca} = 0.25 \sim 0.5\mu M$[8]. Therefore, we assume $K_{s1,IP_3} = 0.4\mu M$. The currents of IP_3 receptors have an obvious decrease at $C_{Ca} = 0.5 \sim 1\mu M$[8]. Then we set $K_{s2,IP_3} = 0.6\mu M$. IP_3 receptors activation and inhibition time scale were adopted by the timescale of Ca^{2+} transients in Merkel cells[9, 10], $\tau_m = 10000ms$, $\tau_h = 20000ms$. The IP_3 receptors open with the increase of IP_3 concentration. $9\mu M$ IP_3 can trigger a saturated open of IP_3 receptors[8], thus we assume $K_{IP_3} = 3\mu M$. IP_3 itself reaches the maximum forming rate at $C_{Ca} = 0.8\mu M$[11]. Then we assume $K_{IP_3,Ca} = 0.5uM$. The concentration

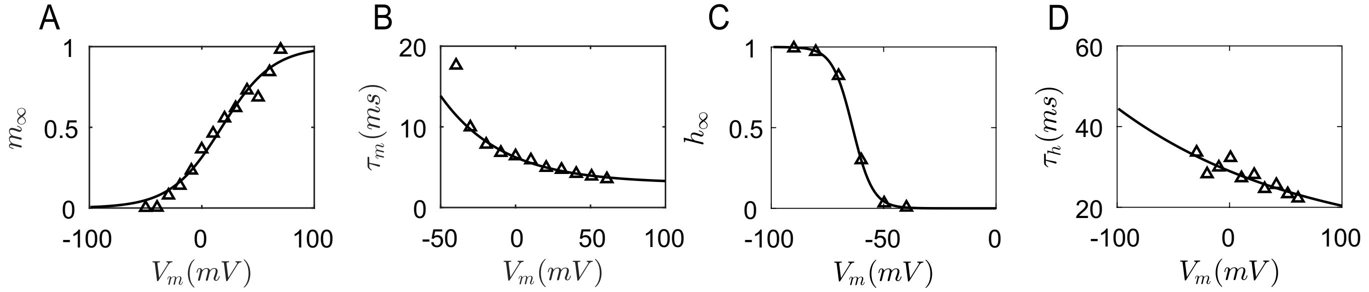

Fig C. $K_v4.2$ channel. (A) Voltage dependence of steady-state activation m_∞ (solid line), triangles represent the experimental data from [6] ($R^2 > 0.97$). (B) Voltage dependence of the activation time constant τ_m (solid line), triangles represent the experimental data from [6] ($R^2 > 0.98$). (C) Voltage dependence of steady-state inactivation h_∞ (solid line), triangles represent the experimental data from [6] ($R^2 > 0.99$). (D) Voltage dependence of the inactivation time constant τ_h (solid line), triangles represent the experimental data from [6] ($R^2 > 0.77$).

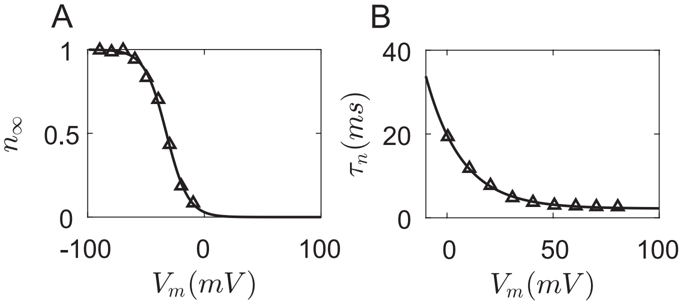

Fig D. KDR channel. (A) Voltage dependence of steady-state activation n_∞ (solid line), triangles represent the experimental data from [6] ($R^2 > 0.99$). (B) Voltage dependence of the activation time constant (τ_n), triangles represent the experimental data from [6] ($R^2 > 0.98$).

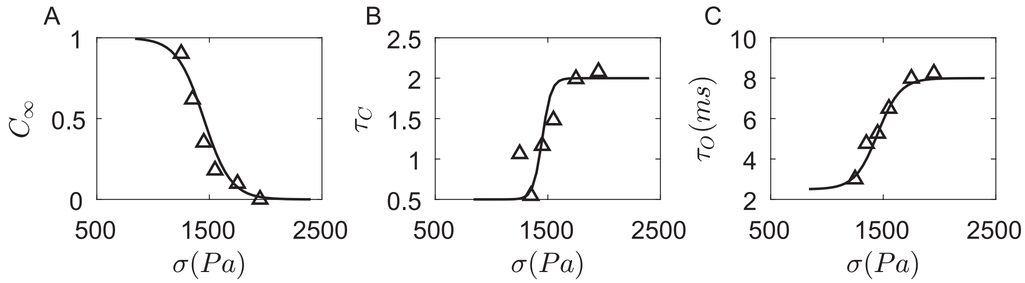

Fig E. $Piezo2$ channel. (A) Cortex stress dependence of the closed state C_∞ (solid line), triangles represent the experimental data for $Piezo2$ channels from [2] ($R^2 > 0.96$). (B) Cortex stress dependence of the closed state time constant (τ_C), triangles represent the experimental data from [2] ($R^2 > 0.71$). (C) Cortex stress dependence of the open time constant (τ_O), triangles represent the experimental data from [2] ($R^2 > 0.92$)

change of IP_3 under Ca^{2+} stimulus is about $1 \sim 10\mu M$[11], which means $preIP_3$ at least has the similar concentration of IP_3. Therefore, we set $K_{s,preIP_3} = 5\mu M$. The concentration of IP_3 reduces to rest in $200 \sim 400s$ with the existing of Ca^{2+}[11], Therefore, we assume $K_{dIP_3} = 2 \times 10^{-5}(1/ms)$ and $K_{IP_3} = 4 \times 10^{-5}(1/ms)$. The area of ER can be estimated from the studies of Halata[12] as $150\mu m^2$, the volume of ER, the surface of MT, and the volume of MT were estimated as $V_{ER} = 100\mu m^3$, $V_{MT} = 150\mu m^2$, $V_{MT} = 10\mu m^3$. The MCU parameters were adopted from experiments of Shutov[13], $K_{MCU} = 0.6\mu M$.

Table 3: The membrane capacitance of Merkel cells is in the range $2 \sim 5pF$[14, 15]. The diameter of Merkel cells is in range $5 \sim 15\mu m$[14–16], of which surface area is about $100 \sim 700\mu m^2$. Then the specific membrane capacitance of Merkel cells C_m lie between $0.3 \sim 5 \times 10^{-8}\mu F/\mu m^2$.

The currents across K^+-relatived channels in Merkel cells I stimulated by voltage clamp range from 300 to $2000pA$[6, 9]. But the currents across one kind of K^+ channels are various. For example, $BKCa$ channels carry $50\% \sim 80\%$ of total K^+ currents in experiments of [9]. While currents across $K_v1.4$ and $K_v4.2$ channels are dominated in total K^+ currents in [6]. The extreme situation is that only one kind of K^+ channel carries the total K^+ currents. The voltage clamp is $120mV$ and the Nerst potential of K^+ channels is about $-80mV$. Then the max conductance of $K_v1.4$ and $K_v4.2$ channels are given by $g_k = I/(S \times (V - E_K)) = 0.3 \sim 2mS/cm^2 (S = 500\mu m^2)$. $BKCa$ channels are also influenced by cytoplasmic Ca^{2+}. Ca^{2+} transients in Merkel cells are more than $1mM$, which are sufficient for $BKCa$ channels to reach the maximum open probability. Then the conductance of $BKCa$ channels is also smaller than $0.3 \sim 2mS/cm^2$. KDR channels in both experiments take up a small part of total K^+ currents, thus the conductance of KDR is smaller than $0.03 \sim 0.2mS/cm^2$.

The flux associated with Na^+/K^+ pumps has been measured to lie between 10^{-16} - $10^{-15} umol \cdot um^2 ms^{-1}$[17]. Dividing this flux by $1/(1 + K_{Na,NaK}/c_{Na})^3 \cdot 1/(1 + C_k/K_{K,NaK})^2 (c_{Na} \approx 14 \times 10^{-12} umol \cdot um^{-3}$ and $c_K \approx 158 \times 10^{-12} umol \cdot um^{-3}$ at rest), $p_{nakpump}$ is ranged from 0.23×10^{-14} to $2.3 \times 10^{-14} umol \cdot um^2 ms^{-1}$, and we set $p_{nakpump} = 0.37312 \times 10^{-14} umol \cdot um^2 ms^{-1} = 0.37312 \times 10^{-12} mol \cdot cm^2 ms^{-1}$.

At rest, K^+ and Ca^{2+} voltage-gated channels are mostly closed. According to the balance of Na^+ and K^+ in the cytoplasm, the leak current of Na^+ and K^+ will be mostly balanced by Na^+/K^+ pumps. $E_{Na} = 60mV$, $E_K = -80mV$, the rest membrane potential is about $-50mV$. Thus $g_{Na,leak} = 0.018 \sim 0.18ms/cm^2$, and $g_{K,leak} =$

$0.05 \sim 0.5ms/cm^2$.

The currents of $KCC2$ are small than $20uA/cm^2$[18, 19]. Divided by $c_{Cl}/c_{Cl,out}c_K/c_{K,out}$, $g_{KCC2} = 2 \times 10^{-13} mol/(cm^2 \cdot ms)$, we get $g_{KCC2} = 0.25 \times 10^{-14} mol/(cm^2 \cdot ms)$. $NKCC1$ usually transports Cl^- into the cell. At rest, Cl^- leak currents dominate the influx of Cl^-. Therefore, we set $g_{NKCC1} = 24cm^{10}/(mol^3 \cdot ms)$, a relatively small value comparing to $g_{Cl,leak}$.

Ca^{2+} currents in Merkel cells are mostly small than $200pA$[6, 9]. The peak currents of Ca^{2+} channels appear at a voltage clamp of $20mV$, and the Nerst potential of Ca^{2+} is about $140mV$. Thus the conductances of $Ca_v2.1$ or $Ca_v2.1$ are smaller than $0.33mS/cm^2$.

Ca^{2+} pumps and Na^+/Ca^{2+} exchangers are responsible for removing Ca^{2+}, and the timescale of this process is much slower than the influx of Ca^{2+} through $Ca_v2.1$ and $Ca_v1.2$. Then the currents of this two channels can be only $0.2pA$, according to the equations of 18 and 19, the $pCapump$ is about $3 \times 10^{-15} mol/(cm^2 \cdot ms)$ and $pCana$ is about $2 \times 10^{-13} mol/(cm^2 \cdot ms)$. At rest, Ca^{2+} leak current is mostly compated with Ca^{2+} pumps, which we set $g_{Ca,leak}$ as $2 \times 10^{-3} mS/cm^2$.

The currents of internal Ca^{2+} haven't direct measurements. But from the results of the inhibition of internal Ca^{2+} stores[9], we can partly estimate the ranges of Ca^{2+} flux in ER. The internal Ca^{2+} stores offer about 2/3 of total Ca^{2+} transients. Therefore, we set $P_{IP_3} = 1.75cm/ms$, and $P_{RYR} = 8.5cm/ms$. Then $P_{pump,ER} = 0.7 \times 10^{-17}$ to balance the outflux of Ca^{2+} from internal Ca^{2+} stores. MCU and MNCX's parameters are adapted from the studies of Mandge[20].

Table 4: Cell membrane area increases by the internal form of vesicles under hypotonic shock. The surface area changes of these cells are ranged from $1000\mu m^2/2min$ to $5000\mu m^2/2min$[21]. A typical vesicle has a radius of $0.1\mu m$. Then the rate of vesicles forming is in the range from $0.06/ms$ to $0.3/ms$. Thus we assume $k_{ve} = 0.1(1/ms)$. Merkel cells contain hundreds or even thousands of vesicles at rest[12]. Therefore we set the controlled parameters of vesicles as 500 and 50. The exocytosis rate has a relative range among cells, especially in neural cells, which can be up to $100 \sim 5000/s$ at peak. Dividing it by vesicle numbers at rest, $k_{exo} = 0.025 \sim 2 \times 10^{-4}(1/ms)$. The rest concentration of Ca^{2+} is about $0.1\mu M$, and $0.2 \sim 0.3\mu M$ of C_{Ca} could cause exocytosis[22]. Then we set the controlled parameters of exocytosis as $0.2\mu M$ and $0.01\mu M$, which means that exocytosis is very sensitive to the concentration of Ca^{2+}. The endocytosis rate also has a wide range. In most cases, its rate will be slower than the corresponding exocytosis rate[23]. Then we assume $k_{endo} = 1(1/ms)$. The controlled parameters of endocytosis were taken from Jiang[24].

The ways of stimuli

At rest, the Merkel cell reaches a balanced state given the initial value of variables (Fig F).

In the cases of Fig 2 and 3,

the current injection $I(t)$ was added to the control equation of membrane potential.

$$C_m \frac{dV_m}{dt} = S_{ref}(J_{K_v1.4} + J_{K_v4.2} + J_{KDR} + J_{BCa} + 2J_{Ca_v1.2} + 2J_{Ca_v2.1} + 2J_{piezo2} - J_{NaKpump} - J_{Cana} - 2J_{Capump} + J_{K,leak} + J_{Na,leak} - J_{Cl,leak} + 2J_{Ca,leak})F + I(t). \quad (10)$$

In the case of Fig 4,

Before stimulation: $C_{Na,out} = 135mM$, $C_{K,out} = 7mM$.

High K^+ solution: $C_{Na,out} = 5mM$, $C_{K,out} = 137mM$.

In the case of Fig 5,

because of adding mannitol to the external solution, the $\Delta\Pi$ was modified as

$$\Delta\Pi = (C_{Na} + C_K + C_{Cl} + C_{Ca} + C_A - C_{Na,out} - C_{K,out} - C_{Cl,out} - C_{Ca,out} - C_{mannitol,out})RT. \quad (11)$$

Before stimulation: $C_{Na,out} = 105mM$, $C_{mannitol,out} = 30mM$.

Hypotonic shock: $C_{Na,out} = 105mM$, $C_{mannitol,out} = 0mM$.

In cases of Fig 6-11, the parameters of the model were based on parameters in Fig 1.

The initial ion concentrations in the external solution are seen in Table B. The initial values of variables in Fig 2 are seen in Table C. If parameters in other simulations change, the model will be first simulated to the new steady state with these initial values before adding stimuli.

The responses of Merkel cells under three kinds of stimuli at the same parameters as shown in Fig H.

All simulations were taken by Matlab, and the code can be seen in zip files.

[1] H. Jiang and S. X. Sun, Biophysical journal **105**, 609 (2013).

Table A. Ion channel parameters in high K^+ solutions and hypotonic shock.

Parameter	Value in high K^+ simulation	Value in hypontonic simulation
$C_m(uF/\mu m^2)$	4×10^{-8}[6]	4×10^{-8}[6]
$g_{K_v1.4}(mS/cm^2)$	0.16[6, 9]	0.16[6, 9]
$g_{K_v4.2}(mS/cm^2)$	0.2[6, 9]	0.2[6, 9]
$g_{BK_{Ca}}(mS/cm^2)$	0.5[6, 9]	0.5[6, 9]
$g_{KDR}(mS/cm^2)$	0.2[6, 9]	0.2[6, 9]
$g_{Ca_v1.2}(mS/cm^2)$	0.04[6, 9]	0.04[6, 9]
$g_{Ca_v2.1}(mS/cm^2)$	0.2×10^{-4}[6, 9]	0.2×10^{-4}[6, 9]
$g_{Piezo2}(mS/cm^2)$	0.3[14]	0.03[14]
$g_{Na,leak}(mS/cm^2)$	0.05 (Tuned)	0.05 (Tuned)
$g_{K,leak}(mS/cm^2)$	0.09 (Tuned)	0.09 (Tuned)
$g_{Cl,leak}(mS/cm^2)$	0.5×10^{-2}(Tuned)	0.5×10^{-2} (Tuned)
$g_{Ca,leak}(mS/cm^2)$	0.2×10^{-4} (Tuned)	0.2×10^{-4} (Tuned)
$P_{NaKpump}(mol/(cm^2\cdot ms))$	0.37312×10^{-12}[17]	0.37312×10^{-12}[17]
$P_{NKCC1}(cm^{10}/(mol^3\cdot ms)$	0.24×10^{2} (Tuned)	0.24×10^{2} (Tuned)
$P_{KCC2}(mol/(cm^2\cdot ms))$	0.25×10^{-14}[18, 19]	0.25×10^{-14}[18, 19]
$P_{Capump}(mol/(cm^2\cdot ms))$	0.3×10^{-15}[10]	0.3×10^{-15}[10]
$P_{Cana}(mol/(cm^2\cdot ms))$	0.3×10^{-13}[9, 10]	0.3×10^{-13}[9, 10]
$P_{pump,ER}(mol/(cm^2\cdot ms))$	0.7×10^{-18}[9, 10]	0.7×10^{-18}[9, 10]
$P_{RYR}(cm/ms)$	0.8×10^{-3} (Tuned)	0.8×10^{-1} (Tuned)
$P_{leak}(cm/ms)$	0.7×10^{-3} (Tuned)	0.7×10^{-3} (Tuned)
$P_{IP_3}(cm/ms)$	14[9, 10]	14[9, 10]
$P_{MCU}(mol/(cm^2\cdot ms))$	0.5×10^{-15} (Tuned)	0.5×10^{-15} (Tuned)
$P_{MNCX}(mol/(cm^2\cdot ms))$	0.1×10^{-15} (Tuned)	0.1×10^{-15} (Tuned)

Table B. Ion concentrations of external solutions

Parameter	Description	Value in simulation
$c_{Na,out}$	external Na^+ concentration (mM)	135[9, 25]
$c_{K,out}$	external K^+ concentration (mM)	7[9, 25]
$c_{Cl,out}$	external Cl^- concentration (mM)	146[9, 25]
$c_{Ca,out}$	external Ca^{2+} concentration (mM)	2[9, 25]

Table C. Initial values of variables

Parameter	initial value in simulation1
$c_{Na}(mM)$	15.76[25]
$c_K(mM)$	156.2[25]
$c_{Cl}(mM)$	6.63[25]
$c_{Ca}(\mu M)$	0.13[25]
$c_A(mM)$	111.4[26]
$V(um^3)$	1216[6]
$c_{IP_3}(\mu M)$	0.42 (Tuned)
$c_{preIP_3}(\mu M)$	3.3 (Tuned)
$c_{Ca,ER}(\mu M)$	700 (Tuned)
$c_{Ca,MT}(\mu M)$	0.2 (Tuned)
$V_m(mV)$	-52.2[6]
$S_{ref}(um^2)$	551[6]

[2] B. Coste, J. Mathur, M. Schmidt, T. J. Earley, S. Ranade, M. J. Petrus, A. E. Dubin, and A. Patapoutian, Science **330**, 55 (2010).
[3] A. H. Lewis, A. F. Cui, M. F. McDonald, and J. Grandl, Cell reports **19**, 2572 (2017).
[4] J. Hao and P. Delmas, Journal of Neuroscience **30**, 13384 (2010).

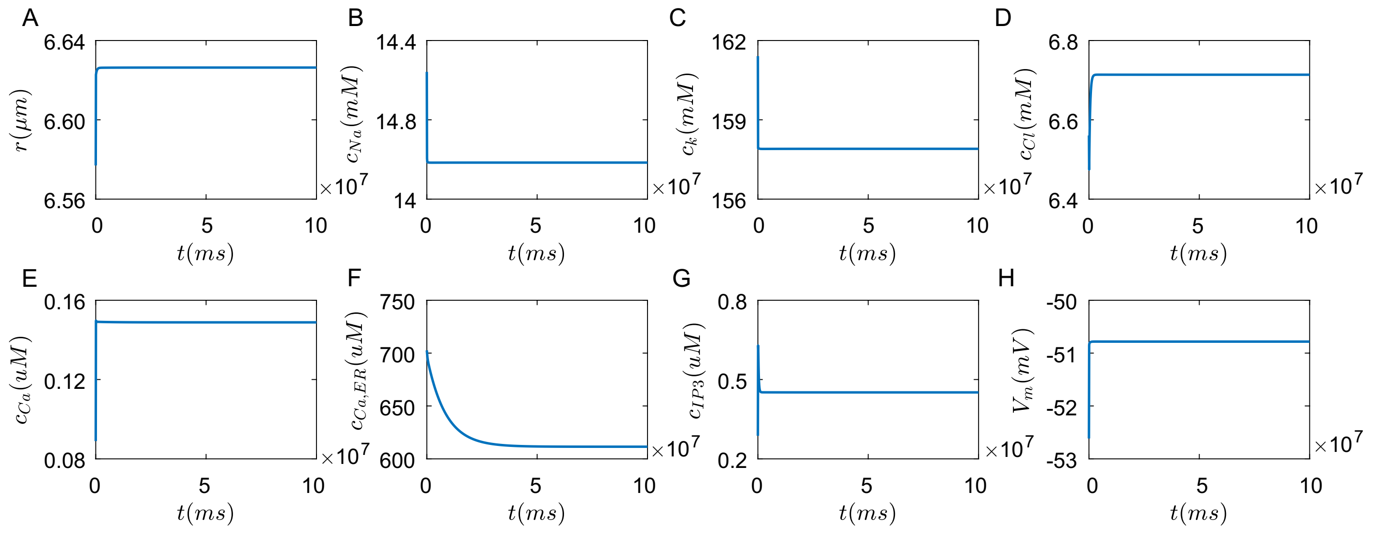

Fig F. Merkel cell reaches a balanced state given an initial value at rest. (A-H) The changes of cell radius(A), intracellular Na^{+}(B), K^{+}(C), Cl^{-}(D), Ca^{2+}(E) concentrations, Ca^{2+} concentration in ER(F), intracellular IP_3 concentration(G) and membrane potential(H) at rest.

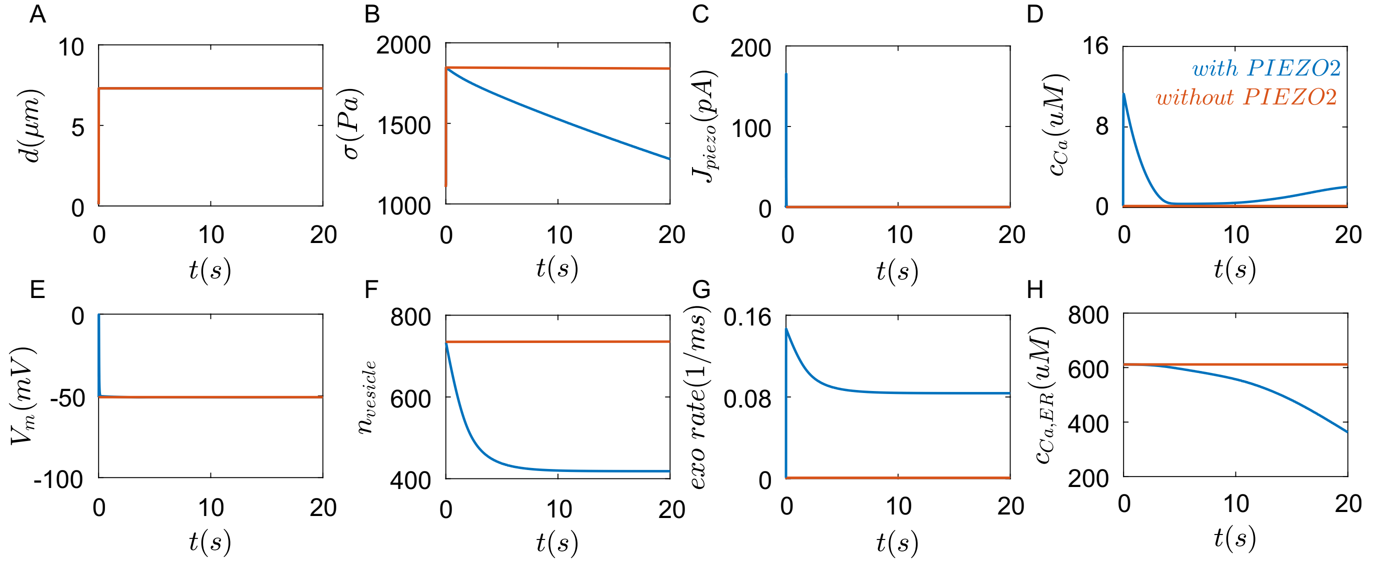

Fig G. The responses of Merkel cells under compression. (A) Compression depth changes with time. (B-H) The dynamic changes of the cortex stress(B), the current across Piezo2 channels(C), intracellular Ca^{2+} concentration(D), membrane potential(E), vesicle number(F), exocytosis rate(G), and concentration of Ca^{2+} in ER(h) (blue: with Piezo2 channels, red: without Piezo2 channels).

[5] J. Roeper, C. Lorra, and O. Pongs, Journal of Neuroscience **17**, 3379 (1997).

[6] Y. Yamashita, N. Akaike, M. Wakamori, I. Ikeda, and H. Ogawa, The Journal of physiology **450**, 143 (1992).

[7] C. C. Fink, B. Slepchenko, I. I. Moraru, J. Watras, J. C. Schaff, and L. M. Loew, Biophysical Journal **79**, 163 (2000).

[8] l. Bezprozvanny, J. Watras, and B. E. Ehrlich, Nature **351**, 751 (1991).

[9] R. Piskorowski, H. Haeberle, M. V. Panditrao, and E. A. Lumpkin, Pflügers Archiv-European Journal of Physiology **457**, 197 (2008).

[10] H. Haeberle, L. A. Bryan, T. J. Vadakkan, M. E. Dickinson, and E. A. Lumpkin, PloS one **3**, e1750 (2008).

[11] K. Hirose, S. Kadowaki, M. Tanabe, H. Takeshima, and M. Iino, Science **284**, 1527 (1999).

[12] Z. Halata, M. Grim, and K. I. Bauman, The Anatomical Record Part A: Discoveries in Molecular, Cellular, and Evolutionary Biology: An Official Publication of the American Association of Anatomists **271**, 225 (2003).

[13] L. P. Shutov, M.-S. Kim, P. R. Houlihan, Y. V. Medvedeva, and Y. M. Usachev, The Journal of physiology **591**, 2443 (2013).

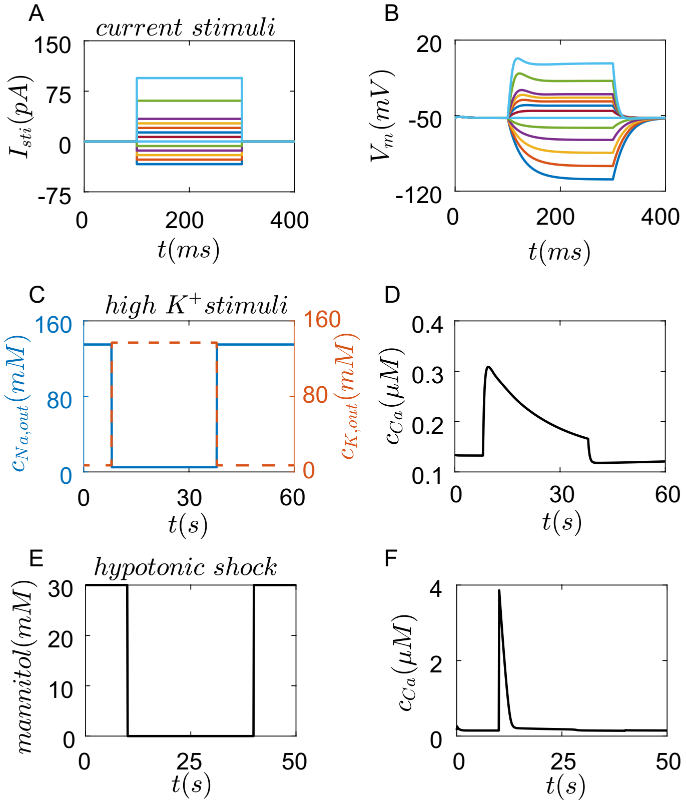

Fig H. The responses of the Merkel cell under three kinds of stimuli with the same parameters. (A) The current stimuli change with time. (B) The dynamic changes of membrane potentials. (C)The Na^{2+} and K^+ concentration changes in external solutions. (D) The dynamic change of intracellular Ca^{2+} concentration. (E)The mannitol cencentration changes in external solutions. (F)The dynamic change of intracellular Ca^{2+} concentration.

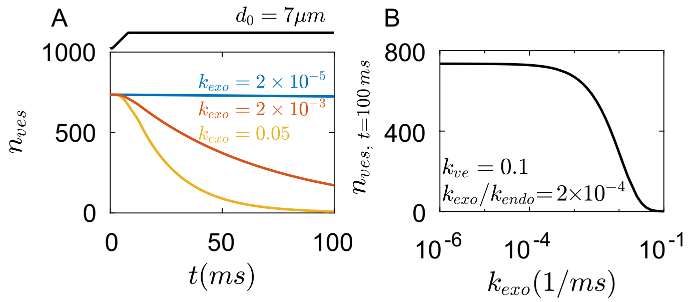

Fig I. The influences of exocytosis rate on vesicle regulation. (A) The dynamic change of vesicles under mechanical stimulus(blue: $k_{exo} = 2 \times 10^{-5}$(1/ms), red: $k_{exo} = 2 \times 10^{-3}$(1/ms), yellow: $k_{exo} = 0.05$(1/ms)). (B) The relation between k_{exo} and vesicle number at $t = 100ms$.

[14] S.-H. Woo, S. Ranade, A. D. Weyer, A. E. Dubin, Y. Baba, Z. Qiu, M. Petrus, T. Miyamoto, K. Reddy, E. A. Lumpkin, *et al.*, Nature **509**, 622 (2014).

[15] R. Ikeda, M. Cha, J. Ling, Z. Jia, D. Coyle, and J. G. Gu, Cell **157**, 664 (2014).

[16] T. Tachibana and T. Nawa, Anatomical science international **77**, 26 (2002).

[17] E. H. Larsen, N. Møbjerg, and R. Nielsen, Comparative Biochemistry and Physiology Part A: Molecular & Integrative Physiology **148**, 101 (2007).

[18] N. Doyon, S. A. Prescott, and Y. De Koninck, Frontiers in cellular neuroscience **9**, 516 (2016).

[19] N. Doyon, L. Vinay, S. A. Prescott, and Y. De Koninck, Neuron **89**, 1157 (2016).

[20] D. Mandge and R. Manchanda, PLoS computational biology **14**, e1006293 (2018).

[21] N. Groulx, F. Boudreault, S. N. Orlov, and R. Grygorczyk, The Journal of membrane biology **214**, 43 (2006).

[22] H. Von Gersdorff and G. Mathews, Nature **367**, 735 (1994).

[23] L.-G. Wu, E. Hamid, W. Shin, and H.-C. Chiang, Annual review of physiology **76**, 301 (2014).

[24] F. Mao, Y. Yang, and H. Jiang, Biophysical Journal **120**, 5521 (2021).
[25] G. B. Ermentrout and D. H. Terman, *Mathematical foundations of neuroscience*, Vol. 35 (Springer Science & Business Media, 2010).
[26] C. M. Armstrong, Proceedings of the National Academy of Sciences **100**, 6257 (2003).
